# Exploring genomic regions involved in bread wheat resistance to leaf rust at seedling/adult stages by using GWAS analysis

**DOI:** 10.1186/s12864-022-09096-1

**Published:** 2023-02-21

**Authors:** Saba Delfan, Mohammad Reza Bihamta, Seyed Taha Dadrezaei, Alireza Abbasi, Hadi Alipour

**Affiliations:** 1 Department of Agronomy and Plant Breeding, Faculty of Agricultural Sciences and Engineering, University of Tehran, Karaj, Iran; 2 Department of Cereal Research, Seed and Plant Improvement Institute, Agricultural Research and Education Organization (AREEO), Karaj, Iran; 3 Department of Plant Production and Genetics, Faculty of Agriculture, Urmia University, Urmia, Iran

**Keywords:** Association mapping, QTL, Rust, Wheat

## Abstract

**Background:**

Global wheat productivity is seriously challenged by a range of rust pathogens, especially leaf rust derived from *Puccinia triticina*. Since the most efficient approach to control leaf rust is genetic resistance, many efforts have been made to uncover resistance genes; however, it demands an ongoing exploration for effective resistance sources because of the advent of novel virulent races. Thus, the current study was focused on detecting leaf rust resistance-related genomic loci against the *P. triticina* prevalent races by GWAS in a set of Iranian cultivars and landraces.

**Results:**

Evaluation of 320 Iranian bread wheat cultivars and landraces against four prevalent rust pathotypes of *P. triticina* (LR-99–2, LR-98–12, LR-98–22, and LR-97–12) indicated the diversity in wheat accessions responses to *P. triticina*. From GWAS results, 80 leaf rust resistance QTLs were located in the surrounding known QTLs/genes on almost chromosomes, except for 1D, 3D, 4D, and 7D. Of these, six MTAs (rs20781/rs20782 associated with resistance to LR-97–12; rs49543/rs52026 for LR-98–22; rs44885/rs44886 for LR-98–22/LR-98–1/LR-99–2) were found on genomic regions where no resistance genes previously reported, suggesting new loci conferring resistance to leaf rust. The GBLUP genomic prediction model appeared better than RR-BLUP and BRR, reflecting that GBLUP is a potent model for genomic selection in wheat accessions.

**Conclusions:**

Overall, the newly identified MTAs as well as the highly resistant accessions in the recent work provide an opportunity towards improving leaf rust resistance.

**Supplementary Information:**

The online version contains supplementary material available at 10.1186/s12864-022-09096-1.

## Background

Wheat as a strategic food crop contributes a quarter of the dietary calories to the global population [1]. Despite this fact, its productivity is seriously influenced by foliar infections, of which rusts such as leaf rust are at the center of pathologists' attention [2]. This disease is derived from *Puccinia triticina* Eriks. (Pt) and gives rise to a remarkable decrease in quality as well as yield [3]. During the early stages of crop growth, this infection leads to more than 50% loss of production [4]. Leaf rust is widespread throughout wheat-growing areas due to its adaptability [5], thus the development of resistant wheat varieties is imperative to protect against yield loss due to leaf rust.

Albeit about eighty genes have been recorded for leaf rust resistance, only some have been successfully exploited in wheat breeding [6, 7]. Most of these genes cause hypersensitive response upon infection due to qualitative race-specific resistance, also called seedling resistance (SR), where the host's resistance and the pathogen's avirulence genes cause incompatible interaction. Such resistance in crops is short-lived because of its failure via a novel virulent race [3]. In contrast, adult plant resistance (APR) genes confer quantitative resistance, which is long-lived, race-non-specific, and controlled by small impact genes, although there are some exceptions, e. g, *Lr13* and *Lr37* are race specific but APR [8]. Most resistance genes confer SR and are race-specific, except for a few genes, such as *Lr68*, *Lr67*, *Lr46*, and *Lr34*, which are race-non-specific and APR [3]. The durability and longevity of leaf rust resistance can be strengthened by a combination of both APR and SR genes [1]. Of course, the long-term use of cultivars with single major *Lr* genes along with the selection pressure on pathogens results in the manifestation of novel races [9]. This highlights the increasing requirement to search for novel resources of resistance genes towards enhancing resistance to emerging races [10]. To date, some researchers have indicated that potential resistance sources can be found in wild relatives, landraces. As a result, such diversities need to be harnessed for identifying new resistance to being used in wheat breeding programs [11].

Albeit plenty of QTLs for leaf rust resistance have been uncovered by linkage mapping, however, there are restrictions related to this mapping, such as low resolution, less diversity sampled, and the long time for establishing a bi-parental population [12, 13]. As an alternative approach, Genome wide association mapping (GWAS) was found helpful for detecting genes/QTLs for wheat resistance to leaf rust. This approach is time-consuming for discovering marker-trait associations (MTAs) or genes/QTLs in natural populations. The uncovering of QTLs in such populations leads to high mapping resolution since they harness all historical recombination events [12]. Various characteristics in wheat, including agro-morphological traits [14], resistance to stripe rust [15], stem rust [16], powdery mildew [17], and fusarium head blight [18] have been dissected successfully by association mapping. There are some reports on mapping QTL/genes involved in bread wheat resistance to leaf rust [9, 19–23], with a potential to marker-assisted selection (MAS).

To apply GWAS, various statistical models, single-locus (MLM) and multi-locus (mrMLM), have been adopted [24–26]. The mrMLM model accounts for large impact loci adequately, whereas the MLM model does not address this issue [25]. The multi-locus model is more reliable than MLM for mapping QTL/genes since all marker impacts are concomitantly estimated and do not need the test of MTAs using rigorous multiple corrections, which leads to the discarding of potential MTAs.

Iran is one of the countries in the Fertile Crescent region, which is known as the center origin and diversity of wheat. Additionally, previous studies have indicated that the center of origin of P. triticina is likely somewhere in the Fertile Crescent region in southwest Asia, where both sexual and asexual reproduction prevail [27]. Therefore, this region could provide an opportunity for natural selection and maintenance of resistance accessions. So, the purposes of this research were to detect genes/QTLs related to leaf rust resistance on a diverse panel of wheat cultivars and landraces originating from several geographical areas in Iran at the adult and seedling growth stages; and compare the resistance genes/QTLs discovered in the recent work with known genes/QTLs.

## Results

### Phenotypic assessment

**Fig. 1:**
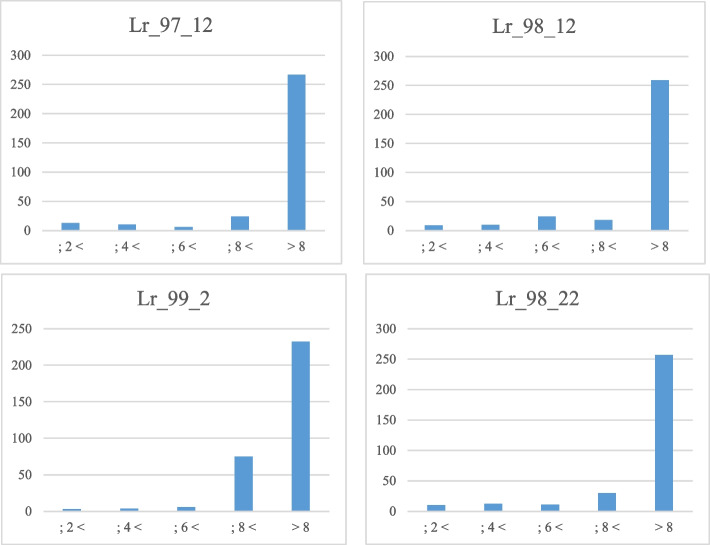
The distribution of phenotypic response of 320 Iranian bread wheat accessions to four leaf rust pathotypes (i.e., LR-97–12, LR-98–12, LR-99–2, and LR-98–22). The x-axis is the linearized disease scale, and the y-axis is the number of wheat accessions

The distribution of phenotypic response of 320 Iranian bread wheat accessions to four leaf rust pathotypes (LR-99–2, LR-98–12, LR-98–22, and LR-97–12) is presented in Fig. 1, suggesting most wheat genotypes are susceptible. Wheat genotypes that are resistant to all four *Puccinia triticina (Pt)* isolates are presented in Table S7. For normality, the SW test indicated a normal distribution for the phenotypic data. Thus, homogeneity within experiments was evaluated by the Levine’s test, which exhibited that the phenotypic variances are homogenous for all pathotypes (*P* = 0.35 to 0.92) (Table S2). As a result, for each wheat accession, the entire mean value was adopted in the association mapping. The H^2^ of the leaf rust ITs ranged from 0.98 to 0.99 for the pathotypes, reflecting most phenotypic diversity can be justified via genotypic variation (Table S2, and S3).

**Fig. 2:**
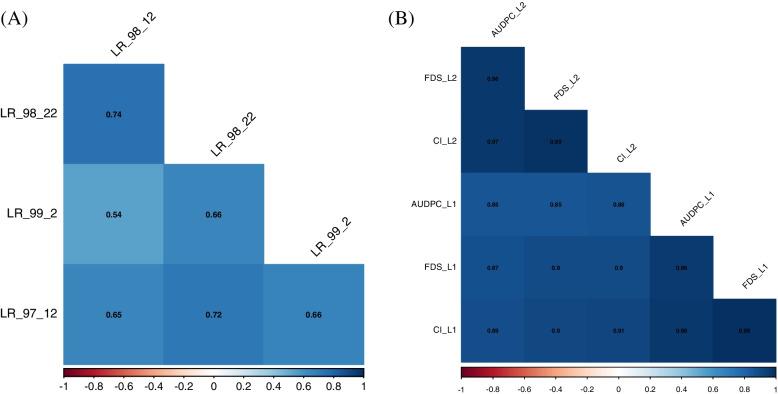
Correlation heatmap and Pearson’s coefficient among pathotypes. **A** At seedling stage and **B** at adult plant stage. L1: Karaj and L2: Ahwaz

From Pearson’s correlation analysis, there are highly significant correlation coefficients (r) among all pathotypes for adult plant and seedling growth stages (Fig. 2). Correlation outcomes indicate that all traits evaluated in the current study (i.e., evaluation parameters of rust infection) can be adopted for association mapping analysis.

The virulence/avirulence profile of the pathotypes’s reactions revealed differences among pathotypes for virulence/avirulence genes (Table S4). Climatic and geographic information for collection locations of wheat leaf rust isolates are presented in Table S5. For pathotypes LR-99–2, LR-98–12, LR-98–22, and LR-97–12, the resistant ITs were recorded in 5, 9.7, 4.4, and 4.4% of the cultivars, respectively, and the resistant ITs in 2.5, 6.6, 7.2, and 6.2% of the landraces, respectively (Table S6). Pathotypes Lr-99–2 and Lr-97–12 were the most virulent on 27.8 and 65.6% of susceptible cultivars and landraces, respectively, while pathotypes Lr-98–12 was the lowest virulent on 22.19 and 60.63% of susceptible cultivars and landraces, respectively. Under field conditions, the adult plant response of the wheat panel was assessed by coefficient of infection (CI), final disease severity (FDS), and area under the disease progression curve (AUDPC) (Table S8). The CI value, which is the product of FDS and AUDPC, was utilized for GWAS analysis. The CI of 20.3 and 23.7% of the wheat genotypes displayed resistance reaction to rust infection in the Ahwaz and Karaj locations, respectively. Furthermore, moderate and susceptible responses were found in 12.5% (Ahwaz) to 24.4% (Karaj) and 67.2% (Ahwaz) to 51.9% (Karaj) wheat genotypes, respectively. Thus, the presence of APR genes in the genetic background of the Iranian wheat accessions could provide resistance in the adult crops.

### Genotypic assessment

From GBS results, a total of 566,439,207 unique reads were recorded with approximately 80% being non-redundant. After de-duplication and alignment, 133,039 SNPs could be called of which 10,938 had a MAF > 1%, H < 10%, and MR < 10% (ensuring quality checks). The ultimate data set consisted of 46,203 imputed SNPs, which were used in GWAS. The highest number of SNPs was detected in the B genome, particularly Chr.6B, Chr.2B, and Chr.3B (Fig. S1).

### Linkage disequilibrium (LD)

The LD value varies both within and between sub-genomes and chromosomes, often decreasing with increasing distance occurring between SNP locations (Table 1). A total of 1,830,925 marker pairs (MP) with *r*^2^ = 0.21 were discovered in wheat cultivars, of which 38% had significant linkages at *P* < 0.001. The highest and lowest numbers of MPs were recorded in the B (51.8%) and D (11.2%) genomes, respectively. The highest LD was found between marker pairs located on the Chr4A, followed by Chr1D (Table 1).

**Table 1:** A summary of observed LD (r^2^) among SNP pairs and the number of significant SNP pairs per chromosomes and genomes of Iranian bread wheat cultivars and landraces

Ch	Cultivar				Landrace			
	TNSP	r^2^	Distance (cM)	NSSP	TNSP	r^2^	Distance (cM)	NSSP
1A	85575	0.148218	1.737691	27125 (31.7%)	92925	0.112764	1.596397	33515 (36.07%)
2A	118025	0.292156	0.974187	57858 (49.02%)	123175	0.297454	0.944378	68675 (55.75%)
3A	83675	0.159365	2.576447	25903 (30.96%)	73525	0.136413	2.939734	28144 (38.28%)
4A	114925	0.371766	1.513597	57774 (50.27%)	108375	0.376224	1.612148	65451 (60.39%)
5A	59375	0.169369	2.383461	18718 (31.53%)	58475	0.150278	2.416511	24007 (41.06%)
6A	85175	0.181387	1.487802	29645 (34.8%)	84425	0.181735	1.501019	40176 (47.59%)
7A	128575	0.234215	1.344495	49426 (38.44%)	126575	0.214252	1.365959	63357 (50.05%)
1B	131075	0.206251	1.063813	49717 (37.93%)	133525	0.157517	1.041252	63803 (47.78%)
2B	165475	0.198105	0.859164	66129 (39.96%)	155625	0.177663	0.913543	78536 (50.46%)
3B	176175	0.245726	0.876581	78363 (44.48%)	170925	0.221549	0.903978	89150 (52.16%)
4B	51325	0.1455	2.516753	13477 (26.26%)	43025	0.1018	3.002768	12311 (28.61%)
5B	134225	0.204683	1.433217	55633 (41.45%)	134675	0.14301	1.449279	56285 (41.79%)
6B	158275	0.205457	0.788418	66108 (41.77%)	164475	0.139023	0.758663	71582 (43.52%)
7B	132875	0.156677	1.102364	41160 (30.98%)	125875	0.129711	1.157535	50573 (40.18%)
1D	37075	0.294821	4.409069	16539 (44.61%)	40975	0.232567	3.832101	19755 (48.21%)
2D	48025	0.23446	2.2455	16275 (33.89%)	52825	0.169092	2.048568	20548 (38.9%)
3D	25475	0.143085	6.286093	5413 (21.25%)	30125	0.174879	5.31564	11411 (37.88%)
4D	10275	0.167587	10.56621	2189 (21.3%)	10375	0.14746	10.71346	3543 (34.15%)
5D	22375	0.155406	9.337668	5503 (24.59%)	24825	0.142184	8.361416	8953 (36.06%)
6D	28475	0.142966	5.369092	6844 (24.04%)	33475	0.14123	4.565844	12606 (37.66%)
7D	34475	0.208327	5.795738	10809 (31.35%)	40475	0.153099	4.947296	14019 (34.64%)
A genome	675325	0.235213	1.620443	266449 (39.4%)	667475	0.223484	1.64269	323325 (48.4%)
B genome	949425	0.20158	1.083656	370587 (39.0%)	928125	0.160951	1.110386	422240 (45.5%)
D genome	206175	0.205106	5.343207	63572 (30.83%)	233075	0.170391	4.707401	90835 (38.97%)
Total	1830925	0.214383	1.761302	700608 (38.3%)	1828675	0.184979	1.76314	836400 (45.7%)

*Abbreviations*: *TNSP* Total number of SNP pairs, *NSSP* Number of significant SNP pairs (*P* value < 0.001)

Applying a similar exploration using the wheat landraces led to detecting 1,828,675 MPs with a mean r^2^ of 0.18, which is lower than in wheat cultivars. A larger fraction of MPs, however, was found to be in significant LD (45.7%). The highest and lowest MPs were registered in the B (928,125) and D (233,075) genomes, respectively. The LD value was found strongest between MPs in the Chr4A, followed by the Chr2A (Table 1).

For estimating LD decay, the LD of 0.157 was determined as the cut-off. It was found that the LD value of the D genome was decayed in a faster manner, followed by the A genome in the wheat accessions panel. In the A genome, the mean LD for MPs was 0.16 at 2.4 Mb in contrast to 1.7 Mb in D chromosomes and 5.4 Mb in the B chromosomes (Fig. S2).

### Population structure

The population structure analysis led to specifying three subpopulations with different levels of admixture (Fig. S3). The population structure matrix revealed the max value of ΔK for K = 3, demonstrating that the Iranian wheat accessions can be divided into three subpopulations.

The PCA was carried out, where PC1 and PC2 justified 16.9 and 6.3% of the diversity variance, respectively. The scatter plot of PCA indicated that the PC1 and PC2 could distinguish the three subpopulations of wheat accessions deriving from various wheat-cultivating areas (Fig. S3), which further supported the outcome of the Structure. Of course, there are some admixed accessions falling between the three subpopulations.

From the cluster analysis of the kinship matrix, the SBP-I subpopulation had 104 accessions (35 landraces and 69 cultivars), and the SBP-II had 108 accessions (102 landraces and 6 cultivars), and the SBP-III had 74 accessions (63 landraces and 11 cultivars) (Fig. S4).

### Marker Trait Association (MTAs)

**Fig. 3:**
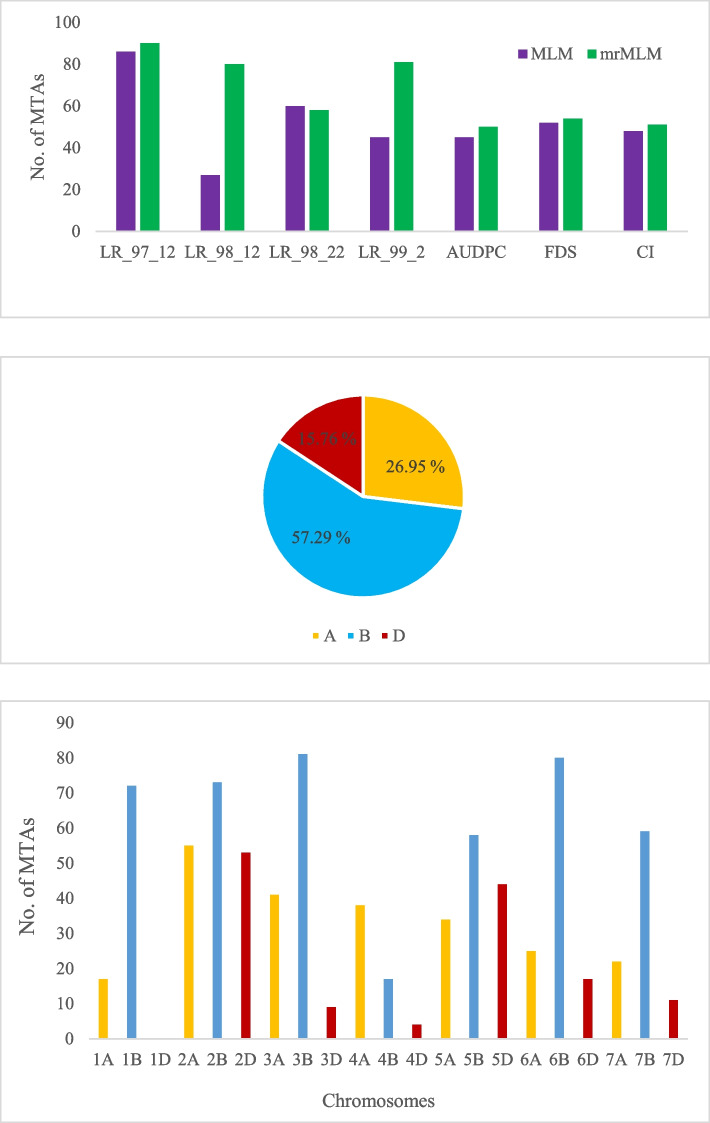
Distribution of the MTAs for resistance to leaf rust. **A** Number of significant MTAs detected for both seedling and adult plant resistance across two GWAS methods. **B** Distribution of the MTAs identified across each genome. **C** Number of MTAs per chromosome

Two GWAS models, MLM and mrMLM, were used to detect defense genomic regions against leaf rust pathotypes at the adult and plant seedling growth stages. As a result, 363 and 464 MTAs were discovered for resistance to various pathotypes by using MLM and mrMLM, respectively (*P* value < 0.001) (Table S9). The mrMLM model appeared the more powerful relative to MLM in our analysis, indicating the max number of highly significant MTAs (74), while MLM was the least potent, as it found the lowest number of highly significant MTAs (39) (Table 2). The highest number of associations was located on the B genome (57.29%) in Chr.1B, Chr.2B, Chr.3B, and Chr.6B (Fig. 3). Exploring both MLM and mrMLM algorithms led to the highest number of MTAs in LR-97–12 pathotype. The phenotypic diversity (R^2^) for both adult and plant seedling growth stages ranged from 2.05% to 3.15%, showing that wheat resistance to pathotypes is modulated by several genomic loci with moderate to small impacts (Table 2).

**Table 2:** Distribution of the highly significant MTAs identified using two GWAS models (*P* value < 0.0001)

No	Trait	Marker	CHR	Allele	Physical position (bp)	R^2^ (%)	Method
1	AUDPC_FDS	rs20790	3B	C/T	55755	2.69	MLM, mrMLM
2	AUDPC_FDS	rs26028	3B	G/T	55755	2.76	mrMLM
3	AUDPC_FDS_CI	rs18695	6D	A/G	55761	2.82	MLM, mrMLM
4	AUDPC_FDS_CI	rs26220	1B	C/G	66042	2.81	MLM, mrMLM
5	AUDPC_FDS_CI	rs26318	2D	A/T	81616	2.79	MLM, mrMLM
6	AUDPC_FDS_CI	rs57400	5B	A/G	26242	2.74	MLM
7	AUPPC_CI	rs2383	2A	C/T	74319	2.54	MLM, mrMLM
8	AUPPC_CI	rs5126	7B	C/T	51193	2.49	MLM, mrMLM
9	CI	rs37116	6B	C/G	43284	2.60	mrMLM
10	CI	rs6872	5B	C/T	127712	2.48	mrMLM
11	CI_FDS	rs39629	3A	G/T	112886	2.79	MLM
12	CI_FDS	rs39631	3A	C/G	112886	2.79	MLM
13	FDS	rs26889	2B	C/G	60320	2.08	MLM, mrMLM
14	FDS	rs39630	3A	T/C	112886	2.76	MLM
15	FDS_CI	rs33218	3B	C/G	54619	2.71	mrMLM
16	LR_97_12	rs13728	7A	A/G	63946	2.61	MLM, mrMLM
17	LR_97_12	rs15875	6B	C/A	85356	2.50	mrMLM
18	LR_97_12	rs15876	6B	A/C	85356	2.49	mrMLM
19	LR_97_12	rs16481	2D	A/T	13642	2.46	mrMLM
20	LR_97_12	rs2007	4A	G/A	124821	2.48	mrMLM
21	LR_97_12	rs2008	4A	C/T	124821	2.48	mrMLM
22	LR_97_12	rs20781	4B	A/G	57770	2.08	mrMLM
23	LR_97_12	rs20782	4B	A/G	57770	2.66	mrMLM
24	LR_97_12	rs24374	6B	T/C	58062	2.54	MLM, mrMLM
25	LR_97_12	rs24375	6B	T/C	58062	2.54	MLM, mrMLM
26	LR_97_12	rs24376	6B	C/T	58062	2.23	MLM, mrMLM
27	LR_97_12	rs32284	1B	A/G	91068	2.57	MLM, mrMLM
28	LR_97_12	rs32285	1B	A/G	91068	2.16	MLM, mrMLM
29	LR_97_12	rs33082	7B	G/A	93505	2.16	mrMLM
30	LR_97_12	rs33083	7B	C/G	93505	2.61	mrMLM
31	LR_97_12	rs34816	5B	A/G	38769	2.50	mrMLM
32	LR_97_12	rs34817	5B	G/A	38769	2.50	mrMLM
33	LR_97_12	rs39680	2B	G/T	66573	2.46	mrMLM
34	LR_97_12	rs41570	6B	C/T	62609	2.69	MLM, mrMLM
35	LR_97_12	rs41571	6B	G/C	62609	2.69	MLM, mrMLM
36	LR_97_12	rs45802	7B	A/C	92919	2.17	mrMLM
37	LR_97_12	rs4602	5B	C/T	35928	2.56	MLM, mrMLM
38	LR_97_12	rs47722	7B	C/T	92332	2.26	mrMLM
39	LR_97_12	rs48776	5B	A/C	62719	2.58	MLM
40	LR_97_12	rs4961	6B	A/G	62609	2.43	mrMLM
41	LR_97_12	rs51004	2B	A/G	66004	2.58	MLM, mrMLM
42	LR_97_12	rs51367	6B	A/T	46694	2.16	MLM, mrMLM
43	LR_97_12	rs57797	6B	A/G	62609	2.53	MLM, mrMLM
44	LR_97_12	rs9211	1B	C/T	46711	2.67	MLM, mrMLM
45	LR_97_12	rs9212	1B	C/T	46711	2.74	MLM, mrMLM
46	LR_97_12, LR_98_22	rs28795	2D	A/G	45240	2.36	mrMLM
47	LR_97_12, LR_98_22	rs53036	2B	A/G	72825	3.06	MLM, mrMLM
48	LR_98_12	rs12518	7B	A/G	50057	2.05	mrMLM
49	LR_98_12	rs25737	3B	C/T	45525	2.65	MLM, mrMLM
50	LR_98_12	rs36084	5B	C/T	45594	2.86	mrMLM
51	LR_98_12	rs36555	6B	G/A	47831	2.20	MLM, mrMLM
52	LR_98_12	rs52542	7B	C/G	114004	2.09	mrMLM
53	LR_98_12	rs58952	5D	G/T	83130	2.08	mrMLM
54	LR_98_12, LR_98_22	rs36483	6B	G/T	94461	3.15	MLM, mrMLM
55	LR_98_12, LR_98_22	rs36484	6B	C/G	94461	3.15	MLM, mrMLM
56	LR_98_12, LR_98_22	rs50047	3B	A/C	22764	3.01	mrMLM
57	LR_98_12, LR_98_22, LR_97_12, LR_99_2	rs7934	3A	C/T	9116	2.62	MLM, mrMLM
58	LR_98_22	rs1798	5D	A/G	83130	2.96	mrMLM
59	LR_98_22	rs1799	5D	A/G	83130	2.96	mrMLM
60	LR_98_22	rs37446	6A	A/G	25146	2.96	mrMLM
61	LR_98_22	rs39305	7B	C/T	43237	3.02	MLM
62	LR_98_22	rs49543	1B	C/T	56943	3.01	mrMLM
63	LR_98_22	rs52026	5A	A/T	93664	2.98	mrMLM
64	LR_98_22	rs52548	2B	C/G	72825	3.02	MLM, mrMLM
65	LR_98_22, LR_98_12, LR_99_2	rs44885	5D	C/T	7959	2.10	mrMLM
66	LR_98_22, LR98_12, LR_99_2	rs44886	5D	A/G	7959	2.20	mrMLM
67	LR_99_2	rs14658	4A	A/G	76969	2.31	mrMLM
68	LR_99_2	rs15675	2B	C/T	6832	2.24	mrMLM
69	LR_99_2	rs15676	2B	C/T	6832	2.24	mrMLM
70	LR_99_2	rs30506	1A	A/G	80900	2.57	MLM, mrMLM
71	LR_99_2	rs38255	3B	A/G	113379	2.25	mrMLM
72	LR_99_2	rs44267	1B	C/G	43301	2.08	mrMLM
73	LR_99_2	rs45341	3B	A/T	113948	2.23	mrMLM
74	LR_99_2	rs62902	6B	C/G	47831	2.57	MLM, mrMLM
75	LR_99_2	rs62903	6B	C/G	47831	2.19	MLM, mrMLM
76	LR_99_2	rs6313	2A	A/G	74319	2.19	mrMLM
77	LR_99_2	rs6314	2A	A/T	74319	2.15	mrMLM
78	LR_99_2	rs750	2B	C/T	67141	2.26	mrMLM
79	LR_99_2	rs9493	3B	A/G	22764	2.85	MLM, mrMLM
80	LR_99_2, LR_97_12	rs59576	2D	A/G	73660	2.26	MLM, mrMLM

### Pleiotropic MTAs

**Fig. 4:**
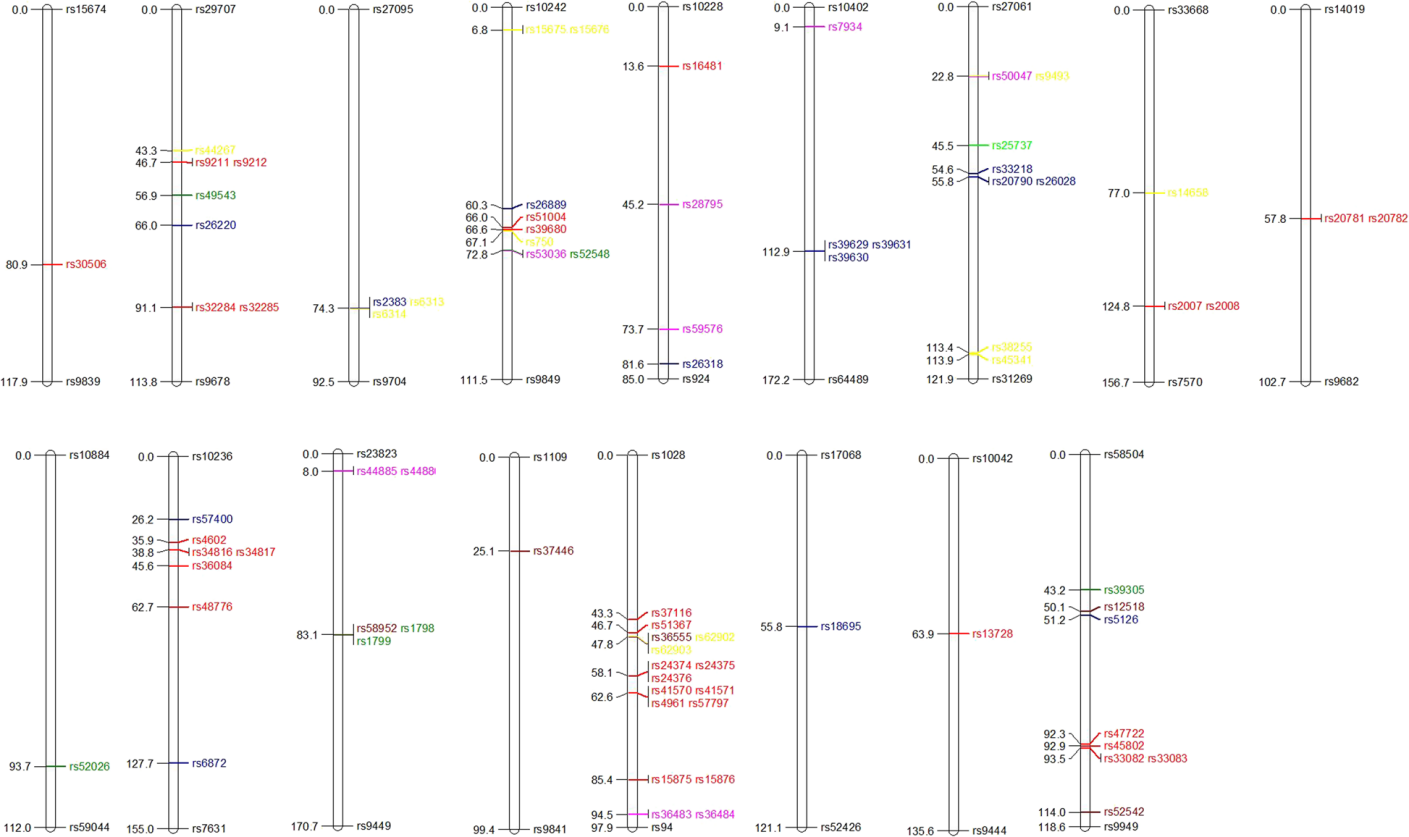
Physical map for seedling and adult plant resistant to leaf rust. Different colors indicate different pathotypes, i.e., red: LR-97–12, brown: LR-98–12, green: LR-98–22, blue: APR, yellow:LR-99–2, pink: two or more pathotypes

Generally speaking, crop response to various pathotypes is connected, and thereby complicated biological mechanisms are responsible for this coordination. In fact, the pleiotropic function of genetic regions on various pathotypes leads to a connection between pathotypes. In this experimental research, some MTAs were observed with pleiotropic impact on the wheat reaction to leaf rust pathotypes (Table 2; Fig. 4). For instance, the MTA rs7934 on Chr.3A was related to all four pathotypes.

### Putative candidate genes

**Fig. 5:**
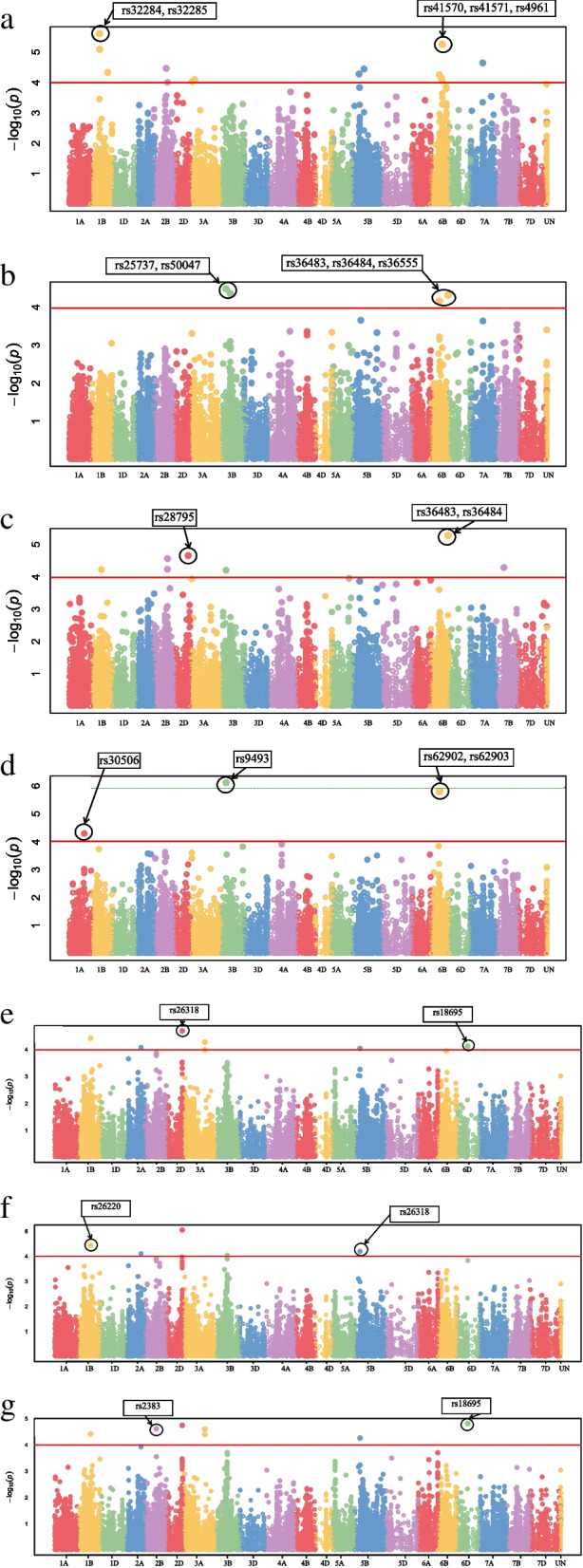
Manhattan plot (showing significant marker-trait associations) for infection type (IT) at seedling stage as well as CI, AUDPC and FDS at adult plant stage to leaf rust. *a* pathotype 97–12, *b* for pathotype 98–12, *c* for pathotype 98–22, *d* for pathotype 99–2, *e* for CI, f for AUDPC and *g* for FDS at adult plant stage

Gene annotation uncovered 80 highly significant MTAs inside protein-coding regions (Table S10), of which 16 reliable MTAs were considered (Table 3). The genes harbouring MTAs mainly encode proteins involved in biological processes in the leaf rust-infected crops, including protein phosphorylation and ubiquitination. The QQ and Manhattan plots of highly associated haplotypes for leaf rust resistance are displayed in Fig. 5. Manhattan plots exhibited significant markers related to resistance to leaf rust pathotypes at *P*-value = 0.0001, as the significant cutoff.

**Table 3:** Candidate genes around the reliable MTAs and their functional annotation (*P* value < 0.0001)

Trait	Marker	Ch	Transcript	Position	Molecular function	Biological Process	Cellular component
LR_99_2	rs30506	1A	TraesCS1A02G031900	_	Protein kinase activity, protein binding, ATP binding	Protein phosphorylation	_
LR_98_22	rs49543	1B	TraesCS1B02G326700	553,017,043–553,022,022	RNA polymerase III general transcription initiation factor activity, DNA binding		Transcription factor TFIIIB complex
LR_98_22	rs52548	2B	TraesCS2B02G040200	18261608–18261643	_	_	Integral component of membrane
LR_99_2	rs750	2B	TraesCS2B02G477000	673961541–673961604	_	_	Integral component of membrane
LR_99_2, LR_97_12	rs59576	2D	TraesCS2D02G479000	580005996–580006059	_	_	
LR_98_12, LR_98_22, LR_97_12, LR_99_2	rs7934	3A	TraesCS3A02G013400	9783188–9783251	Protein kinase activity, ATP binding	Protein phosphorylation	_
LR_97_12	rs2007	4A	TraesCS4A02G441100	709857646–709857703	_	_	_
LR_98_12	rs36084	5B	TraesCS5B02G273100	458663129–458663192	_	_	_
LR_98_22	rs1798	5D	TraesCS5D02G412500	475,691,763–475,695,452	Catalytic activity	_	_
LR_97_12	rs41571	6B	TraesCS6B02G453300	711,709,311–711,712,023	Protein binding		_
LR_97_12	rs47722	7B	TraesCS7B02G407200	676249917–676249980	Magnesium ion binding, terpene synthase activity, lyase activity		_
LR_97_12	rs39680	2B	U6	646960537–646960600	_	_	_
CI	rs6872	5B	TraesCS5B02G501400	668,581,998–668,583,715	Aspartic-type endopeptidase activity	_	Integral component of membrane
CI	rs37116	6B	TraesCS6B02G125200	120318178–120318241	Protein binding	SCF-dependent proteasomal ubiquitin-dependent protein catabolic process	SCF ubiquitin ligase complex
AUPPC_CI	rs5126	7B	TraesCS7B02G225900	426526075–426526138	Protein binding		
LR_97_12	rs45802	7B	TraesCS7B02G407200	676249917–676249980	Magnesium ion binding, terpene synthase activity, lyase activity

### Genomic prediction (GP)

**Fig. 6:**
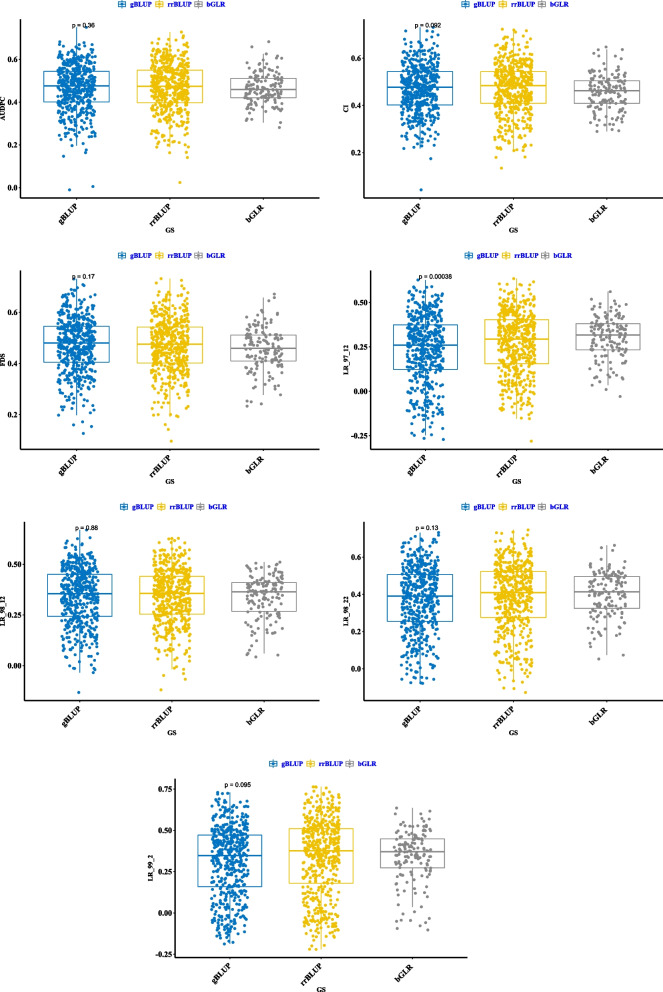
The impact of genomic selection (GS) methodologies on GP accuracy for leaf rust resistance in Iranian landraces and cultivars. The GP for RR-BLUP, GBLUP, and BGRR is presented with blue, orange and gray colors, respectively. The boxplots exhibit the first, second, and third quartile. The middle points show a mean of GP accuracies for the trait of interest

The highest prediction accuracy was achieved for the GBLUP approach (Fig. 6). Overall, the genomic best linear unbiased prediction (GBLUP) model appeared better than ridge regression-best linear unbiased prediction (RR-BLUP) and bayesian ridge regression (BRR), highlighting that it is the preferable algorithm to use for genomic selection in the Iranian wheat panel.

## Discussion

Exploring new resources of resistance QTLs/genes is an ongoing task and is imperative in crop improvement for combating the pathogen threat to productivity. These genes or QTLs can be pyramided towards the development of durable resistant varieties. To achieve these goals, GWAS is used successfully in wheat to discover genomic regions involved in leaf rust resistance at the adult plant and seedling stages [3, 28]. Therefore, this study was aimed at detecting resistance genes/QTLs/markers against leaf rust in Iranian bread wheat accessions.

### Phenotypic variation for wheat resistance to leaf rust

Iranian wheat cultivars and landraces were screened for resistance to leaf rust at the adult plant and seedling stages under field and in a controlled situation, respectively. The crop panel exhibited a diversity in the leaf rust response in both growth stages. The distribution of leaf rust phenotypic reactions skewed toward susceptibility in seedlings and toward resistance in adult crops, showing the existence of APR genes in the Iranian wheat. This reflects the importance of APR genes for rust resistance in breeding endeavors, however, APR in wheat natural populations is interesting as well. The APR genomic regions among natural populations, new or known, diversify resistance to leaf rust. From our observations, wheat landraces were found more resistant to Lr-98–22 when compared to cultivars. Exploring landraces for harnessing new alleles responsible for leaf rust resistance is also made by other studies [20, 23], that emphasized on the importance of natural populations to find resistance sources for leaf rust.

From correlation analysis, significant relations were found between the infection types (ITs) of *P. triticina* pathotypes. This high correlation may originate due to several factors. The first evidence comes from a virulence profiling of wheat varieties harboring the *Lr* gene that exhibited all *P. triticina* races were virulent to *Lr14a, Lr11*, *Lr3*, and *Lr1*[21]. Thus, it is expected correlations among the phenotypic data. Second evidence, the association panel in the current work likely had common QTLs conferring resistance to *P. triticina* pathotypes and this was verified further by association mapping, which allowed the discovery of common QTLs involved in resistance to all four pathotypes. A similar observation was observed earlier, where a high correlation was recorded in the phenotypic reactions with multiple *P. triticina* races, followed by detecting common QTLs for resistance to those races [21].

### Population structure

Since genetic variation is the critical factor for crop improvement endeavors [29], assessment of the population structure is a requirement for using a genetic resource in wheat manipulation [30]. From our observations, different analysis approaches agreed with the presence of three subpopulations and also consent with the geographic origin. Structure analysis revealed three subpopulations among the 320 Iranian wheat accessions and the outcome from the PCA also supports this grouping. Interestingly, the clustering pattern of wheat accessions was not in line with their origins or geographical distributions. It seems that this is likely due to the farmers’ migration and germplasm exchange across institutes [3].

### LD of marker pairs

A total of 18,932 SNPs was recorded on wheat chromosomes, especially in genome B, which possesses the highest density of markers, followed by the A and D genomes. A similar pattern has been also reported by others [31, 32]. The higher variation in the B and A wheat genomes is likely the consequence of two factors [33], the older evolutionary history of these genomes, and gene flow from the species *T. turgidum* (but not *Ae. tauschii*) to common wheat. The fact that wheat cultivars show higher LD in contrast to landraces is likely the consequence of selection events during crop breeding practices. Overall, mating systems, recombination, genetic drift, population relatedness, mutation, and selection are all major elements influencing LD in wheat [34]. The LD decay rate was more rapid in the D genome than in others, which is in line with earlier reports [15].

### MLM and mrMLM

The mrMLM model appeared the more powerful relative to MLM in our analysis, indicating the max number of highly significant associations, while MLM was the least potent, as it found the lowest number of highly significant associations. MLM single-locus model adopts a genome scan test one SNP at a time while needing multiple corrections (e.g., Bonferroni) for avoiding false positives. This process is too conservative and may lead to the loss of actual associations that are fundamental to the intended characteristic [25. Moreover, single-locus models cannot simultaneously estimate all marker impacts, and thereby cannot present a proper model for genetic mapping the quantitative properties, which are governed by the cumulative act of numerous genes [25]. For overcoming these challenges, the mrMLM multi-locus approach was also adopted for dissecting the molecular basis of stress tolerance in wheat [12].

### Putative candidate genes

GWAS is a well-known molecular approach in crop breeding programs to discover MTAs related to pathogen resistance. To date, this approach has been utilized successfully in plants to find genomic regions involved in leaf rust resistance at the adult plant and seedling stages [23, 28]. Our attempt led to detecting a total of 80 highly significant MTAs and 16 reliable MTAs for leaf rust resistance. The distribution of these MTAs was found cross 21 chromosomes except for Chr1D, Chr3D, Chr4D, and Chr7D. Earlier experimental evidence has also detected MTAs on almost 21 chromosomes [35], as both minor as well as major genes be involved in leaf rust resistance during adult plant and seedling. For example, it has been stated there are not any MTAs related to seedling/adult leaf rust resistance on Chr6A, Chr5B, Chr4D, Chr4B, and Chr4A on wheat accessions [36].

The location of MTAs detected in the recent work was compared with those of previous reports and the results were presented at Table S11. We must remind that for some of the MTAs, it is a difficult comparison across various studies because of the difference in the mapping population and marker platforms, as well as the absence of a consensus map for comparing MTA locations [1]. Also, the reliable comparison is possible only on the basis of physical map information, for example, the rs52548 MTA located on the long arm of 2B at 72.825 Mb region was determined to be highly significant for LR-98–22 resistance, and is near the QLr.stars-2BL1, Lr50, and Lr58 markers [37–39].

In this experimental research, the rs7934 pleiotropic MTA was to influence the wheat reaction to four leaf rust pathotypes. Similarly, it has been observed four pleiotropic QTLs for APR in a Chinese collection of wheat, including QLr-5BL/QYr-5BL.1, QLr-2AL.2/QYr-2AL.2, QLr-2AL.1/QYr-2AL.1, and Lr46/Yr29 [19]. Such loci affecting several pathotypes and two growth stages can be used as promising markers for MAS after passing the validation process.

From functional point of view, most genes responsible for leaf rust resistance included those coding proteins involved in Mg^2+^ ion binding, terpene synthase activity, lyase activity, aspartic-type endopeptidase activity, etc. For instance, the putative candidate gene related to seedling resistance to LR-97–12 pathotype belongs to the protein serine/threonine kinases, which have a key function in disease resistance and recognition of pathogens [15]. In a similar research attempt, exploring MTAs related to seedling/adult leaf rust resistance on a panel of 400 wheat accessions led to the identification of candidate genes, such as serine-threonine and leucine-rich repeat receptor-like protein kinases, and P-loop containing nucleoside triphosphate hydrolases, which have a function in disease resistance and pathogen recognition [36]. The flanking SNP closely linked to leaf rust resistance can be converted to allele-specific markers in MAS to deliver these genomic regions into wheat breeding lines [4].

There are three families of R genes in wheat crops, depending on durability, specificity, as well as sensitivity [40]: *i*) the genes encode the START proteins [STaR-related lipid-transfer] family and are specific versus all races of one pathogenic species, i.e. confer non-race-specific resistance; *ii*) the genes encode proteins belonging to ATP-binding cassettes [ABC] family such as *Lr34* and induce non-race-specific resistance versus several races of more than one pathogen; *iii*) the genes encode proteins belonging to NLR family [NBD and leucine-rich repeat] and induce race-specific resistance versus one but not to other races of the same pathogenic species [3]. Interestingly, some SNPs were in significant association with *Lr* genes *Lr1* (rs58952 associated with resistance to LR-98–12; rs1798/rs1799 for LR-98–22), *Lr2* (rs16481 for LR-97–12), *Lr3* (rs15875/rs15876 for LR-97–12; rs36483/rs36484 for LR-98–12/LR-98–22), *Lr9* (rs37116 for CI; rs51367 for LR-97–12; rs36555 for LR-98–12; rs62902/rs62903 for LR-99–2), *Lr13* (rs15675/rs15676/rs15675/rs15676 for LR-99–2), *Lr15* (rs16481 for LR-97–12), *Lr16* (rs15675 for LR-99–2), *Lr22* (rs16481 for LR-97–12), *Lr23* (rs15675 for LR-99–2), *Lr27* (rs50047 for LR-98–12/LR-98–22; rs9493 for LR-99–2), *Lr38* (rs2383 for AUDPC-CI; rs6313/rs6314 for LR-99–2), *Lr39* (rs16481 for LR-97–12), *Lr48* (rs15675 for LR-99–2), *Lr50* (rs39680/rs51004/rs53036 for LR-97–12; rs52548 for LR-98–22; rs750 for LR-99–2), *Lr52* (rs57400 for AUDPC-FDS-CI traits; rs34817/rs34816/rs4602 for LR-97–12; rs36084 for LR-98–12), *Lr54* (rs59576 for LR-99–2/LR-97–12), *Lr58* (rs39680/rs51004/rs53036 for LR-97–12; rs52548 for LR-98–22; rs750 for LR-99–2), *Lr63* (rs7934 for four races), *Lr66* (rs7934 for four races), *Lr68* (rs33082/rs33083/rs45802/rs47722 for LR-97–12; rs52542 for LR-98–12), *Lr73* (rs15675 for LR-99–2). However, it should remind that although the examined isolates show virulence against most of the mentioned *Lr* genes, perhaps minor effects, independent of virulence, are still detectable. From the known genomic locations of *Lr* resistant genes in bread wheat [1], some MTAs such as rs58952-*Lr1* on 5D were in line with previous reports and some such as rs2383-Lr38 on 2A were in contrast. Thus, the MTAs discovered in this research further require to be validated before using in wheat programs.

Among the ~ 80 *Lr* genes, most genes provide SR, and only a few genes such as *Lr77*, *Lr68*, *Lr67, Lr46, Lr34*, *Lr22, Lr13,* and *Lr12* have been reported to harbor APR [41]. After passing the validation process, the markers associated with *Lr13, Lr22,* and *Lr68* genes in this study can be used to pyramid the APR genes and improve leaf rust resistance in wheat high-yielding cultivars. Moreover, annotation of genes for APR uncovered the most similar proteins responsible for SR since both share the elicitor responses and signalizing cascades. A meta-analysis explored consensus genomic regions conferring wheat resistance to leaf rust by using 393 QTLs collected from 50 QTL mapping reports [3]. The finding was the detecting of 15 high confidence meta-QTLs, which can be used in MAS. For example, MQTL7B.3 is co-localized with both *Lr14a* and *Lr68* genes. The SR gene *Lr14a* is presumed to have originated from emmer wheat and is related to resistance genes *Lr68* and *Sr17* (for powdery mildew as well as stem rust). Moreover, *Lr14a* can confer APR to most *P. triticina* races with low-to-medium ITs as well as is connected with necrosis. As a result, the MQTL7B.3 not only induces SR as well as APR but also provides a region of resistance to multiple diseases.

Of highly significant 80 QTLs detected in the surrounding known QTLs or genes, six MTAs (rs20781 and rs20782 for LR-97–12; rs49543 and rs52026 for LR-98–22; rs44885 and rs44886 for LR-98–22, LR-98–1, LR-99–2) were found on genomic regions where no Lr genes have been reported in wheat, reflecting new QTLs or genes for leaf rust resistance.

### Genomic prediction

Generally speaking, the GP accuracy depends on several factors, including the LD levels, genetic diversity in populations, genomic selection methodology, and the genetic architecture of the trait [42]. In the recent study, we found that GBLUP appeared as better than RR-BLUP and BRR, reflecting that GBLUP is a potent model for implementing genomic selection in wheat accessions. From earlier studies, high prediction accuracy can be obtained by GBLUP if SNPs are tightly linked to the intended trait [12]. RR-BLUP works well for crop traits where the genetic architecture consists of multiple loci with small impacts, while the BRR is similar to RR-BLUP, except marker impact shrinkage depends on the size of the population in BRR. The better performance of GBLUP depends on the fact that SNPs in the recent work were closely linked with wheat resistance to leaf rust.

## Conclusions

The current study was focused on 320 Iranian bread wheat cultivars and landraces against four prevalent races of *P. triticina* in Iran. GWAS analysis led to discovering 80 highly significant and 16 reliable MTAs for leaf rut resistance on almost all chromosomes. Among these, six QTLs including rs20781 and rs20782 (for LR-97–12), rs49543 and rs52026 (for LR-98–22), as well as rs44885 (for LR-98–22, LR-98–1, and LR-99–2) and rs44886 (for LR-98–22, LR-98–12, LR-99–2) were found on genomic regions where no *Lr* genes previously reported in wheat, suggesting new loci for leaf rust resistance. Other QTLs were uncovered in the adjacency of previously reported *Lr* QTLs/genes, thus, further analysis such as an allelism test is needed to specify their association. The *rs44885 and rs44886* MTAs appeared to be reliable for resistance to all pathogenic races with the main impact and altogether justified up to 6% of the observational diversity.

## Methods

### Plant and pathogen materials

A total of 320 Iranian bread wheat accessions were obtained from the seed collection of University of Tehran -Agriculture and Natural Resources (UT-ANR), and the Seed and Plant Improvement Institute (SPII), Karaj, Iran (Table S1). The evaluation of the desired genotypes in the adult and seedling stages against the dominant pathotypes of leaf rust in the country (LR-99–2, LR-98–12, LR-98–22, and LR-97–12) was carried out in the cereal pathology greenhouse of the SPII. The field experiment with the common pathotypes of Khuzestan province was conducted at the Ahvaz station and the common pathotypes of Alborz province at the research field of UT-ANR. The study protocol must comply with relevant institutional, national, and international guidelines and legislation.

### Seedling resistance evaluation

Cultivation practices and recording of the response of wheat accessions to the rust were carried out in the spring. The accessions were cultivated in the form of a randomized complete block design (RCBD), which was implemented with two replicates. In all experiments, for each accession, seven seeds were planted in a pot (containing peat moss, sand, and field soil) and kept in greenhouse conditions (at 21 °C and 65% relative humidity). Irrigation was also done by the leakage method. About 8–10 days after sowing the seeds, with the completion of the first leaf, the seedlings were ready for inoculation. Inoculation was done by using a mix of brown rust spores and Talc powder in a ratio of 1:4 by using a brush. After inoculation, the pots were kept for 24 h in a dark and cold room at 17 °C and saturated relative humidity. The pots then were transferred to greenhouses at 22 °C, photoperiod of 16/8 h of light/darkness, and relative humidity of 75%. About 10 days after inoculation, the infection types created on the wheat accessions were recorded based on a scale of 0 to 4 as follows [43]: O or immune, without any visible marks and pustules; H or hypersensitivity, the appearance of hypersensitive flecks in the form of necrosis and chlorosis without pustules; 1 or resistant, small pustules covered by necrotic spots; 2 or semi-resistant, small to moderate pustules covered by chlorosis or necrosis; 3 or susceptible, moderate-sized pustules that may be associated with chlorosis; 4 or very susceptible, small pustules without chlorosis and necrosis. Finally, the 0–4 scale was converted to 0–9 numerical scales, scores in the range of 0–7 were considered as resistant and above 7 as susceptible [44].

### Adult plant stage evaluation

In the Ahvaz location, the genotypes were cultivated in the middle of December 2018 in the field research of Khuzestan Agriculture and Natural Resources, Ahvaz, Iran (latitude: 31˚20ꞌN, longitude: 48˚40ꞌE, and height from the sea level: 13 m). Each of the wheat accessions was cultivated on a one-meter line with a distance of 30 cm. After ten accessions, the susceptible variety Bolani was cultivated as a control. This susceptible variety was also cultivated around the experimental as the spreader of the disease and its over-receiver. This experiment was evaluated under sprinkler irrigation, which allows the application of water under high pressure with the help of a pump. Artificial inoculation was done using a mixture of brown rust pathotypes of the region (in equal proportion of each pathotype) starting from January 10^th^ and until February 25^th^, once every 15 days, in the evening. After the uniformity of the appearance of the disease on the susceptible variety (Bolani), the severity of the flag leaf infection was recorded by using determining the percentage of leaf surface contamination (0–100%) [45] and also by determining the IT [46] on four occasions with 10-day intervals from the 6th of March. Scoring was as follows [44]: O or immune, without any symptoms; R or resistant, the appearance of chlorotic and necrotic band spots without pustules, or small and scattered pustules; MR or semi-resistant, the appearance of small rust pustules surrounded by necrotic spots; MS or semi- susceptible, the appearance of medium-sized pustules, without necrotic spots, sometimes with chlorotic spots; S or susceptible, the appearance of large rust pustules in abundance without chlorotic spots, sometimes with these spots.

When the disease severity in the susceptible variety Bolani reached 100%, the last recording (4^th^) in wheat accessions was considered as the final severity of infection FDS. The severity of the disease in each note-taking and its host reaction (infection types including R or resistant, MR or semi-resistant, MS or semi- susceptible, and S or susceptible) were combined to calculate the CI. That is, the CI was obtained from the product of the disease severity and the constant-coefficient related to the host reaction (S = 1, MS = 0.8, MR = 0.4, *R* = 0.2, and 0 = 0). From the obtained CI, the area under the disease progression curve (AUDPC) was estimated as follows:

$${A}_{k}=\sum_{i=1}^{{N}_{i}-1}\frac{({y}_{i}+{y}_{i+1})}{2} \left({t}_{i+1}-{t}_{i}\right)$$

where t_i_, recording time t_i_; *t*_*i*+1_ − *t*_*i*_: recording time *t*_*i*_ + 1 m; y_i_: rust infection coefficient at the time of recording t_i_; *y*_*i*+1_: rust infection rate at the time of recording *t*_*i*+1_; N: the number of records to assess the disease severity.

In the Karaj location, the genotypes were cultivated in the middle of November 2019 in the research field of University of Tehran (latitude: 36˚00ꞌN, longitude: 48˚40ꞌE, and height from the sea level: 1137 m). Each of the wheat accessions was cultivated on 2 m lines with a distance of 30 cm. Artificial inoculation was done using a mixture of brown rust pathotypes of the region (in equal proportion of each pathotype) starting from March 30^th^ and until May 15^th^, once every 15 days, in the evening. After the uniformity of the appearance of the disease on the susceptible variety (Bolani), the severity of the flag leaf disease was recorded by using determining the percentage of leaf surface contamination (0–100%) [45] and also by determining the infection type [46] on four occasions with 10-day intervals from the 6th of March.

### Statistical analysis

The phenotypic data were analyzed via SAS V 9.4. Pearson’s correlation was estimated using the corrplot package in R. For determining if the phenotypic data for each race was normally distributed, the Shapiro–Wilk (SW) test was carried out using the Proc-Univariate procedure. Moreover, for checking the data homogeneity, Levene’s test was conducted. The entire mean was utilized for the association analysis if the data were homogenous. To calculate H^2^ (broad-sense heritability), genotypic variation was divided by the sum of error, block, and genotypic variances.

### Genotyping and SNP imputation

To genotype Iranian bread wheat cultivars and landraces by genotyping-by-sequencing (GBS), the GBS library was developed and sequenced [47, 48]. BLAST, which permits for mismatches up to 3 bp, was exploited to discover SNPs. These polymorphisms were called by TASSEL using the UNEAK pipeline [49]. To avoid false-positive SNPs, only those with a missing rate (MR) < 10% across samples, heterozygosity (H) < 10%, and minor allele frequencies (MAF) > 1% were included. Missing data were imputed via TASSEL using the LD KNNi [49]. As the reference genome, the W7984 bread wheat genome was adopted to call SNPs [50].

### Population genetic analysis

Population structure was determined using a mixed model in the Structure [51]. The number of subpopulations (K) varied from K = 1 to K = 10 and for each value, ten independent runs of 10,000 burn-in and 10,000 MCMC steps were done. The most likely K value was specified via the ΔK in the Structure harvester [52]. The matrix of population structure, i.e. Q, was obtained for the whole population from the Structure analysis for the best value of K [49]. The kinship matrix (K) was also obtained using the EMMA in the R [53]. To support the Structure outcomes, a principal component analysis (PCA) was applied to the SNPs using the Tidyverse in the R [50].

### LD

From the value of expected/observed allele frequencies, LD occurred among SNPs was estimated in TASSEL V.5. The pairwise LD was calculated using the squared correlation coefficient of alleles (r^2^). The full matrix option was used to estimate the LD distribution for each subpopulation and in the whole panel. From the theoretical expectation of r^2^, LD decay was determined for each chromosome and genome [54].

### GWAS analysis

Association mapping was implemented using MLM single-locus [26] and mrMLM multi-locus [25]. Briefly, the MLM procedure was conducted by MLM package procedure, while the mrMLM using the mrMLM package in R [25] in two steps. First, all potentially associated SNPs were included in a second model where their impacts were estimated using an empirical Bayes approach. Finally, a likelihood ratio test was used to evaluate all non-zero marker impacts. A significance threshold (cut-off) of − log_10_ (*P*-value) ≥ 3.0 (*P* ≤ 0.001) was considered for detecting significant associations in the model. All SNPs meeting this cut-off value were categorized as significant MTAs. GWAS outcomes were summarised using Manhattan plots to visualize associations between accessions and traits in the package GAPIT [55]. In this plot, the x-axis and y-axis present the genomic positions of SNPs and the − log_10_ (*P*-value) obtained from the F-test, respectively. A Q-Q plot was also performed to test the distribution of *P*-values [56].

### Identification of candidate genes

Surrounding all highly significant SNPs, genome sequences were collected and exploited for gene annotation with BLAST versus the IWGSC RefSeq v1.0 genome reference for wheat [50]. After alignment, genes with the highest blast score and identity were included. The biological processes and molecular functions of putative genes were found in Ensembl Plants [http://plants.ensembl.org]. The discovery of candidate genes was tested based on being located in the vicinity of the peak marker with a domain 1 Mb.

### GP

The genomic prediction in this study was carried out using three models: RR-BLUP [57], GBLUP [58], and BRR [59]. All analyses for GP were implemented using the iPat software [60]. For three sub-populations, 10, 20, and 30% of accessions were assigned randomly to a validation set with the remaining individuals used as the training set. For all of the GP procedures, the whole prediction process was repeated 100 times for each method. The prediction accuracy was represented as the correlation between BLUPs and GEBVs over the validation as well as training sets [12].

## Supplementary Information

**Additional file 1: Table S1.** List of 320 Iranian wheat accessions.

**Additional file 2: Table S2.** Basic statistics of the phenotypic data of 320 wheat genotypes evaluated for their reaction to four leaf rust pathotypes. **Table S3.** Analysis of variance for Infection type reactions of wheat genotypes (320 accessions) against leaf rust isolates. **Table S4.** Avirulence and virulence profile of leaf rust pathotypes. **Table S5.** Climatic and geographic information for collection locations of wheat leaf rust isolates.**Table S6**. Classification of wheat genotypes (320 accessions) based on the reaction to four Pt races (The infection types on the wheat accessions are based on McIntosh et al., (1995) procedure). **Table S7.** List of wheat genotypes that are resistant to all four Puccinia triticina (Pt) isolates (infection-type reactions are based on Macintosh et al (1985) procedure). **Table S8.** Leaf rust response of wheat accessions at the adult plant stage.**Table S9.** Details of 201 and 65 significant MTAs detected for both seedling and adult plant resistance to leaf rust-associated pathotypes using two GWAS methods (mrMLM and MLM) (*P* value < 0.001). **Table S10.** Candidate genes around the reliable MTAs and their functional annotation for seedling and adult plant resistance. **Table S11.** Comparison of most significant identified QTLs with previously published *Lr* genes or QTLs for both seedling and adult plant resistance to leaf rust [61–81].

**Additional file 3: Fig S1.** Distribution of SNPs on three genomes (A) and on each chromosome (B). **Fig. S2.** LD decay plot of the (a) A genome, (b) B genome, (c) D genome, and (d) whole genome. Genetic distance in cM is plotted against the LD estimate (*r*2) for pairs of markers. The blue horizontal line indicates R2 threshold (R2=0.1), the green vertical line indicates LD decay distance, and the red line is the moving average of the 10 adjacent markers. **Fig. S3.** Structure plot of 320 Iranian bread wheat accessions determined by K=3 (A) and Principal component analysis (B).Fig. S4 Cluster analysis using kiniship matrix for Iranian wheat accessions.

## Data Availability

All data generated or analyzed during this study are included in this published article.

## Abbreviations

*Pt*: *Puccinia triticina* Eriks
SR: Seedling resistance
APR: Adult plant resistance
GWAS: Genome wide association mapping
MTA: Marker-trait associations
MAS: Marker-assisted selection
LD: Linkage disequilibrium
MP: Marker pairs
GP: Genomic prediction
IT: Infection type
FDS: Final disease severity
CI: Coefficient of infection
AUDPC: Area under the disease progression curve
RR-BLUP: Ridge regression-best linear unbiased prediction
GBLUP: Genomic best linear unbiased prediction
BRR: Bayesian ridge regression
