# Correction to: Exploring genomic regions involved in bread wheat resistance to leaf rust at seedling/adult stages by using GWAS analysis

**DOI:** 10.1186/s12864-023-09360-y

**Published:** 2023-05-17

**Authors:** Saba Delfan, Mohammad Reza Bihamta, Seyed Taha Dadrezaei, Alireza Abbasi, Hadi Alipour

**Affiliations:** 1 Department of Agronomy and Plant Breeding, Faculty of Agricultural Sciences and Engineering, University of Tehran, Karaj, Iran; 2 Department of Cereal Research, Seed and Plant Improvement Institute, Agricultural Research and Education Organization (AREEO), Karaj, Iran; 3 Department of Plant Production and Genetics, Faculty of Agriculture, Urmia University, Urmia, Iran

**Correction to**: ***BMC Genomics*24**, 83 (2023).

10.1186/s12864-022-09096-1

Following the publication of the original article [1], the authors identified an error in the author name Hadi Alipour.

The incorrect author’s name is: Hadi Alipoor.

The correct author’s name is: Hadi Alipour.

The author group has been updated above and the original article [1] has been corrected.
