# Supplementary material for: Exploring genomic regions involved in bread wheat resistance to leaf rust at seedling/adult stages by using GWAS analysis
Source: BMC Genomics. 2023 Feb 21;24:83. doi: 10.1186/s12864-022-09096-1 (PMC9945389; doi:10.1186/s12864-022-09096-1)
Supplement: Supplementary file 2 — Additional file 2: Table S2. Basic statistics of the phenotypic data of 320 wheat genotypes evaluated for their reaction to four leaf rust pathotypes. Table S3. Analysis of variance for Infection type reactions of wheat genotypes (320 accessions) against leaf rust isolates. Table S4. Avirulence and virulence profile of leaf rust pathotypes. Table S5. Climatic and geographic information for collection locations of wheat leaf rust isolates.Table S6. Classification of wheat genotypes (320 accessions) based on the reaction to four Pt races (The infection types on the wheat accessions are based on McIntosh et al., (1995) procedure). Table S7. List of wheat genotypes that are resistant to all four Puccinia triticina (Pt) isolates (infection-type reactions are based on Macintosh et al (1985) procedure). Table S8. Leaf rust response of wheat accessions at the adult plant stage.Table S9. Details of 201 and 65 significant MTAs detected for both seedling and adult plant resistance to leaf rust-associated pathotypes using two GWAS methods (mrMLM and MLM) (P value < 0.001). Table S10. Candidate genes around the reliable MTAs and their functional annotation for seedling and adult plant resistance. Table S11. Comparison of most significant identified QTLs with previously published Lr genes or QTLs for both seedling and adult plant resistance to leaf rust [61–81]. [file 12864_2022_9096_MOESM2_ESM.docx]

**Table S2**: Basic statistics of the phenotypic data of 320 wheat genotypes evaluated for their reaction to four leaf rust pathotypes.

| Pathotype | Race^a^ | Mean | Min | Max | SD | Shapiro_Wilk test^b^ | Leven’s test^c^ | H^2^ |
| --- | --- | --- | --- | --- | --- | --- | --- | --- |
| LR_97_12 | FFRSQ | 8.30 | 0 | 9.00 | 1.87 | P<0.0001 | P=0. 35 | 0.98 |
| LR_98_12 | PFTTT | 8.12 | 0.35 | 9.00 | 1.88 | P<0.0001 | P=0.917 | 0.99 |
| LR_98_22 | PKTTT | 8.23 | 0.35 | 9.00 | 1.81 | P<0.0001 | P=0.863 | 0.97 |
| LR_99_2 | PDKTT | 8.37 | 1.19 | 9.00 | 1.11 | P<0.0001 | P=0.779 | 0.99 |

SD-Standard Deviation, H^2^-Broad-sense heritability.

|  |  | MS (Mean of Squares) | | | |
| --- | --- | --- | --- | --- | --- |
| S. O. V | df | Lr_98_12 | Lr_97_12 | Lr_99_2 | Lr_98_22 |
| Block | 1 | 1.08^ns^ | 0.08^ns^ | 0.35^ns^ | 0.27^ns^ |
| Genotype | 319 | 7.07^***^ | 8.01^***^ | 2.48^***^ | 6.52^***^ |
| Error | 319 | 0.08 | 0.20 | 0.22 | 0.24 |
| CV (%) |  | 6.52 | 4.85 | 5.26 | 7.12 |

Table S3. Analysis of variance for Infection type reactions of wheat genotypes (320 accessions) against leaf rust isolates

S. O. V: Source of Variance; df: Degree of Freedom; CV: Coefficient of Variation

^ns^, and ^**^ non-significant and significant at 0.1% probability levels, respectively

**Table S4:** Avirulence and virulence profile of leaf rust pathotypes.

| No. | Pathotypes | Location | Source of collection | Race | *Virulence genes* | *Avirulence genes* |
| --- | --- | --- | --- | --- | --- | --- |
| 1 | LR-97-12 | Khorram abad_Lorestan | *Triticum aestivum* L. | FFRSQ | *Lr22b, Lr2c, Lr3, Lr3ka, Lr3bg, Lr10, Lr11, Lr12, Lr13, Lr14a, Lr14b, Lr15, Lr22a, Lr23, Lr24, Lr25, Lr26, Lr29, Lr30, Lr32, Lr33, Lr34, Lr35, Lr36, Lr37, Lrb, Lr13* | *Lr1, Lr2a, Lr2b, Lr9, Lr16, Lr17, Lr18, Lr19, Lr20, Lr21, Lr10 / Lr27 + / Lr31, Lr28* |
| 2 | LR-98-12 | Shavoor_Khouzestan | *Triticum aestivum* L. | PFTTT | *Lr22b, Lr1, Lr2b, Lr2c, Lr3, Lr3ka, Lr3bg, Lr10, Lr11, Lr12, Lr13, Lr14a, Lr14b, Lr15, Lr17, Lr18, Lr20, Lr21, Lr22a, Lr23, Lr24, Lr25, Lr26, Lr10 / Lr27 + / Lr31, Lr28, Lr29, Lr30, Lr32, Lr33, Lr34, Lr35, Lr36, Lr37, Lrb, Lr13* | *Lr2a, Lr9, Lr16, Lr19* |
| 3 | LR-98-22 | Sistan Balouchestan | *Triticum aestivum* L. | PKTTT | *Lr22b, Lr1, Lr2c, Lr3, Lr3ka, Lr3bg, Lr10, Lr11, Lr12, Lr13, Lr14a, Lr14b, Lr15, Lr16, Lr17, Lr18, Lr20, Lr21, Lr22a, Lr23, Lr24, Lr25, Lr26, Lr28, Lr30, Lr32, Lr33, Lr34, Lr35, Lr36, Lr37, Lrb, Lr13* | *Lr2a, Lr2b, Lr9, Lr19, Lr10 / Lr27 + / Lr31, Lr29* |
| 4 | LR_99_2 | Ahwaz_Khouzestan | *Triticum aestivum* L. | PDKTT | *Lr22b, Lr1, Lr2c,Lr3, Lr3bg, Lr10,(Lr10, Lr27+Lr31), Lr11, Lr12, Lr13, Lr14a, Lr14b, Lr15,Lr16, Lr17, Lr18, Lr20, Lr21, Lr22a, Lr23, Lr24, Lr25, Lr28, Lr30, Lr32, Lr33, Lr34, Lr35, Lr36, Lr37, Lrb* | *Lr2a, Lr2b, Lr3ka, Lr9, Lr16, Lr19, Lr26, (Lr10, Lr27+Lr10), Lr29* |

Table S5. Climatic and geographic information for collection locations of wheat leaf rust isolates

| Isolates | Location | Collection year | Max temperature (°C) | Min temperature (°C) | Average temperature (°C) | Total rainfall (mm) | Latitude | Longitude | Height (m) |
| --- | --- | --- | --- | --- | --- | --- | --- | --- | --- |
| LR-99-2 | Ahwaz_Karaj | 2019-2020 | 33.9 | 19 | 26.6 | 240.59 | 31˚20ꞌN | 48˚40ꞌE | 13 |
| LR-97-12 | Khorram abad | 2019-2020 | 25.4 | 9.4 | 17.5 | 801.37 | 33˚30ꞌN | 48˚25ꞌE | 1393 |
| LR-98-12 | Shavvoor | 2020-2021 | 32.8 | 16.5 | 24.4 | 396.23 | 32˚20ꞌN | 48˚30ꞌE | 120 |
| LR-98-22 | Sistan balachestan | 2019-2020 | 31.7 | 27 | 22.1 | 168.55 | 28˚46ꞌN | 53˚52ꞌE | 2043 |

Table S6   Classification of wheat genotypes (320 accessions) based on the reaction to four Pt races (The infection types on the wheat accessions are based on McIntosh et al., (1995) procedure)

|  | Cultivar/  Landrace | Lr-97-12 | | Lr_98_12 | | Lr_98_22 | | Lr_99_2 | |
| --- | --- | --- | --- | --- | --- | --- | --- | --- | --- |
| Infection Type |  | Number | % | Number | % | Number | % | Number | % |
| Resistance reaction | | | | | | | | | |
| 0 and ; | Cultivar | 1 | 0.31 | 0 | 0 | 1 | 0.31 | 0 | 0 |
|  | Landrace | 0 | 0 | 3 | 0.94 | 0 | 0 | 0 | 0 |
| ; 1, 1 and 1+ | Cultivar | 1 | 0.31 | 0 | 0 | 0 | 0 | 0 | 0 |
|  | Landrace | 4 | 1.25 | 4 | 1.25 | 4 | 1.25 | 2 | 0.63 |
| ; 2, 2 and 2+ | Cultivar | 3 | 0.94 | 12 | 3.75 | 7 | 2.19 | 0 | 0 |
|  | Landrace | 1 | 0.31 | 8 | 2.5 | 5 | 1.56 | 2 | 0.63 |
| ; 1 2 3 | Cultivar | 11 | 3.44 | 19 | 5.94 | 6 | 1.88 | 14 | 4.38 |
|  | Landrace | 3 | 0.94 | 6 | 1.88 | 14 | 4.38 | 16 | 5 |
| Total | Cultivar | 16 | 5 | 31 | 9.69 | 14 | 4.38 | 14 | 4.38 |
|  | Landrace | 8 | 2.5 | 21 | 6.56 | 23 | 7.19 | 20 | 6.25 |
| Susceptible reactions | | | | | | | | | |
| 3 and 3+ | Cultivar | 56 | 17.5 | 71 | 22.19 | 80 | 25 | 87 | 27.19 |
|  | Landrace | 149 | 46.56 | 196 | 61.25 | 196 | 61.25 | 190 | 59.38 |
| 4 | Cultivar | 30 | 9.38 | 1 | 0.31 | 2 | 0.63 | 2 | 0.63 |
|  | Landrace | 61 | 19.06 | 0 | 0 | 5 | 1.56 | 7 | 2.19 |
| Total | Cultivar | 86 | 26.88 | 71 | 22.19 | 82 | 25.63 | 89 | 27.81 |
|  | Landrace | 210 | 65.63 | 194 | 60.63 | 201 | 62.81 | 197 | 61.56 |
| Total accessions |  | 320 | 100 | 320 | 100 | 320 | 100 | 320 | 100 |

| Accession | Origin | LR_98_12 | LR_98_22 | LR_99_2 | LR_97_12 |
| --- | --- | --- | --- | --- | --- |
| 627189 | Khorasan_Bojnourd | ; 3 | ; 3 | 3 ; | 2+ ; |
| 627856 | Mazandaran_Sari | ; 1 | 1 ; | ; 1 | ; 1 |
| 627963 | Hamedan_Hamedan | ; 1 2 | ; 1 | ; 3 | ; 1 |
| 622084 | Mazandaran_Sari | ; 1 | ; 2 | 3 ; | ; 2+ |
| 622099 | Gilan_Rasht | ; 1 | ; 2 | ; 2 | ; 2+ |
| 622247 | Mazandaran_Sari | ; 1 | ; 1 | 3 ; | ; 1 |
| 622264 | Mazandaran_Babol | ; 1 | 1 ; | ; 1+ | ; 1 |
| 622272 | Mazandaran_Amol | 6.67 | ; 1 | ; 2+ | ; 1 |
| 624381 | Bakhtaran_Bakhtaran | ; 2 | 1 ; | ; 2 | ; 1 |
| RASHID | _ | ; 2+ | ; 2 | ; 3 | ; 2 |

Table S7. List of wheat genotypes that are resistant to all four *Puccinia triticina (Pt)* isolates (infection-type reactions are based on Macintosh et al (1985) procedure)

**Table S8:** Leaf rust response of wheat accessions at the adult plant stage.

| **Reaction** | **Number of accessions (Percentage)** | | | | | |
| --- | --- | --- | --- | --- | --- | --- |
|  | **CI** | | **FDS** | | **AUDPC** | |
|  | Ahwaz | Karaj | Ahwaz | Karaj | Ahwaz | Karaj |
| **Resistant** | 65 (20.31%) | 76 (23.75%) | 49 (15.31%) | 75 (23.44%) | 39 (12.19%) | 74 (23.13%) |
| **Moderate** | 40 (12.5%) | 78 (24.38%) | 56 (17.5%) | 79 (24.69%) | 25 (7.81%) | 29 (9.06%) |
| **Susceptible** | 215 (67.19%) | 166 (51.88%) | 215 (67.19%) | 166 (51.88%) | 256 (80%) | 217 (67.81%) |

CI: Coefficient of Infection

FDS: Final Disease Severity

AUDPC: Area Under the Disease Progress Curve

**Table S9:** Details of 201 and 65 significant MTAs detected for both seedling and adult plant resistance to leaf rust-associated pathotypes using two GWAS methods (mrMLM and MLM) (P value < 0.001)

| Trait | Marker | CHR | Allele | position (cM) | R^2^ | Method | |
| --- | --- | --- | --- | --- | --- | --- | --- |
| AUDPC | rs13744 | 6A | A/G | 99.391 | 2.71 |  | MLM |
| AUDPC | rs16458 | 2A | C/T | 59.228 | 2.69 |  | MLM |
| AUDPC | rs16459 | 2A | G/C | 59.228 | 2.69 |  | MLM |
| AUDPC | rs24881 | 1B | A/C | 32.984 | 2.70 |  | MLM |
| AUDPC | rs32494 | 3B | A/G | 65.99 | 2.68 |  | MLM |
| AUDPC | rs3724 | 1B | C/T | 32.984 | 2.74 |  | MLM |
| AUDPC | rs39629 | 3A | G/T | 112.886 | 2.68 |  | MLM |
| AUDPC | rs39631 | 3A | C/G | 112.886 | 2.68 |  | MLM |
| AUDPC | rs50039 | 3B | C/T | 54.619 | 2.70 |  | MLM |
| AUDPC | rs50947 | 5B | A/T | 18.219 | 2.67 |  | MLM |
| AUDPC | rs5185 | 2D | A/C | 81.616 | 2.73 |  | MLM |
| AUDPC | rs5936 | 1A | A/G | 68.388 | 2.73 |  | MLM |
| AUDPC | rs59987 | 1B | A/C | 36.398 | 2.68 |  | MLM |
| AUDPC | rs61673 | 6B | G/T | 17.12 | 2.67 |  | MLM |
| AUDPC | rs9286 | 6A | C/T | 99.391 | 2.67 |  | MLM |
| AUDPC | rs9382 | 2B | A/G | 77.383 | 2.66 |  | MLM |
| AUDPC | rs9410 | 3B | A/G | 40.978 | 2.72 |  | MLM |
| AUDPC | rs13733 | 2A | C/T | 10.254 | 2.74 | MLM | mrMLM |
| AUDPC | rs15134 | 2A | C/T | 59.228 | 2.59 |  | mrMLM |
| AUDPC | rs17332 | 1B | A/G | 88.795 | 2.55 |  | mrMLM |
| AUDPC | rs18379 | 1B | C/T | 75.148 | 2.68 | MLM | mrMLM |
| AUDPC | rs18695 | 6D | A/G | 55.761 | 2.77 | MLM | mrMLM |
| AUDPC | rs20790 | 3B | A/G | 55.755 | 2.80 | MLM | mrMLM |
| AUDPC | rs20851 | 6B | C/T | 47.263 | 2.72 | MLM | mrMLM |
| AUDPC | rs21979 | 1B | C/T | 113.814 | 2.70 | MLM | mrMLM |
| AUDPC | rs23749 | 7B | A/G | 115.14 | 2.66 | MLM | mrMLM |
| AUDPC | rs2383 | 2A | C/T | 74.319 | 2.81 | MLM | mrMLM |
| AUDPC | rs23886 | 6B | C/T | 43.284 | 2.67 | MLM | mrMLM |
| AUDPC | rs24174 | 3B | A/T | 54.619 | 2.66 | MLM | mrMLM |
| AUDPC | rs24786 | 1B | A/G | 66.042 | 2.60 |  | mrMLM |
| AUDPC | rs24788 | 1B | G/A | 66.042 | 2.62 |  | mrMLM |
| AUDPC | rs26028 | 3B | C/T | 55.755 | 2.78 | MLM | mrMLM |
| AUDPC | rs26220 | 1B | G/T | 66.042 | 2.86 | MLM | mrMLM |
| AUDPC | rs26318 | 2D | C/G | 81.616 | 2.94 | MLM | mrMLM |
| AUDPC | rs26889 | 2B | A/T | 60.32 | 2.77 | MLM | mrMLM |
| AUDPC | rs2720 | 2D | A/C | 81.616 | 2.75 | MLM | mrMLM |
| AUDPC | rs28342 | 1B | G/T | 66.042 | 2.62 |  | mrMLM |
| AUDPC | rs28343 | 1B | C/T | 66.042 | 2.62 |  | mrMLM |
| AUDPC | rs28447 | 2D | A/T | 81.616 | 2.79 | MLM | mrMLM |
| AUDPC | rs30894 | 7B | G/A | 51.193 | 2.58 |  | mrMLM |
| AUDPC | rs32500 | 2D | A/G | 81.616 | 2.77 | MLM | mrMLM |
| AUDPC | rs33218 | 3B | C/G | 54.619 | 2.68 | MLM | mrMLM |
| AUDPC | rs34317 | 6A | C/G | 53.619 | 2.71 | MLM | mrMLM |
| AUDPC | rs34865 | 2B | A/G | 111.506 | 2.51 |  | mrMLM |
| AUDPC | rs34866 | 2B | A/C | 111.506 | 2.51 |  | mrMLM |
| AUDPC | rs37116 | 6B | C/G | 43.284 | 2.71 | MLM | mrMLM |
| AUDPC | rs41930 | 5B | C/T | 38.769 | 2.58 |  | mrMLM |
| AUDPC | rs44265 | 3B | A/G | 54.619 | 2.66 |  | mrMLM |
| AUDPC | rs5126 | 7B | C/T | 51.193 | 2.63 |  | mrMLM |
| AUDPC | rs54172 | 3B | A/G | 56.892 | 2.55 |  | mrMLM |
| AUDPC | rs57076 | 2D | C/G | 81.616 | 2.76 | MLM | mrMLM |
| AUDPC | rs57124 | 6B | A/G | 92.187 | 2.63 |  | mrMLM |
| AUDPC | rs57400 | 5B | A/G | 26.242 | 2.82 | MLM | mrMLM |
| AUDPC | rs5799 | 2B | C/T | 77.383 | 2.74 | MLM | mrMLM |
| AUDPC | rs5800 | 2B | T/C | 77.383 | 2.76 | MLM | mrMLM |
| AUDPC | rs58019 | 2A | A/G | 87.971 | 2.51 |  | mrMLM |
| AUDPC | rs58596 | 5B | C/G | 109.918 | 2.52 |  | mrMLM |
| AUDPC | rs58765 | 2B | A/G | 60.32 | 2.78 | MLM | mrMLM |
| AUDPC | rs59033 | 4A | A/C | 62.152 | 2.41 |  | mrMLM |
| AUDPC | rs59523 | 1A | C/T | 3.415 | 2.67 |  | mrMLM |
| AUDPC | rs60746 | 3B | A/G | 54.619 | 2.64 |  | mrMLM |
| AUDPC | rs6166 | 2A | C/G | 74.319 | 2.63 |  | mrMLM |
| AUDPC | rs6456 | 2B | C/G | 60.32 | 2.66 | MLM | mrMLM |
| AUDPC | rs6872 | 5B | C/T | 127.712 | 2.53 |  | mrMLM |
| AUDPC | rs8636 | 4A | G/T | 62.152 | 2.45 |  | mrMLM |
| AUDPC | rs9504 | 3A | A/G | 10.254 | 2.68 | MLM | mrMLM |
| AUDPC | rs9745 | 4B | C/T | 70.847 | 2.58 |  | mrMLM |
| CI | rs12012 | 2A | A/G | 74.319 | 2.52 |  | mrMLM |
| CI | rs13733 | 2A | C/T | 10.254 | 2.66 | MLM | mrMLM |
| CI | rs13744 | 6A | A/G | 99.391 | 2.58 |  | MLM |
| CI | rs15134 | 2A | C/T | 59.228 | 2.51 | MLM | mrMLM |
| CI | rs16458 | 2A | C/T | 59.228 | 2.59 |  | MLM |
| CI | rs16459 | 2A | G/C | 59.228 | 2.59 |  | MLM |
| CI | rs17332 | 1B | A/G | 88.795 | 2.47 | MLM | mrMLM |
| CI | rs18379 | 1B | C/T | 75.148 | 2.61 | MLM | mrMLM |
| CI | rs18695 | 6D | A/G | 55.761 | 2.73 | MLM | mrMLM |
| CI | rs20790 | 3B | A/G | 55.755 | 2.64 | MLM | mrMLM |
| CI | rs20851 | 6B | C/T | 47.263 | 2.60 | MLM | mrMLM |
| CI | rs21979 | 1B | C/T | 113.814 | 2.63 | MLM | mrMLM |
| CI | rs23749 | 7B | A/G | 115.14 | 2.54 |  | mrMLM |
| CI | rs2383 | 2A | C/T | 74.319 | 2.72 | MLM | mrMLM |
| CI | rs24174 | 3B | A/T | 54.619 | 2.60 | MLM | mrMLM |
| CI | rs24786 | 1B | A/G | 66.042 | 2.49 |  | mrMLM |
| CI | rs24788 | 1B | G/A | 66.042 | 2.53 |  | mrMLM |
| CI | rs25504 | 5D | C/T | 33.541 | 2.65 | MLM | mrMLM |
| CI | rs26028 | 3B | C/T | 55.755 | 2.64 | MLM | mrMLM |
| CI | rs26220 | 1B | G/T | 66.042 | 2.77 | MLM | mrMLM |
| CI | rs26318 | 2D | C/G | 81.616 | 2.81 | MLM | mrMLM |
| CI | rs26889 | 2B | A/T | 60.32 | 2.68 | MLM | mrMLM |
| CI | rs2720 | 2D | A/C | 81.616 | 2.61 |  | MLM |
| CI | rs28342 | 1B | G/T | 66.042 | 2.53 |  | mrMLM |
| CI | rs28343 | 1B | C/T | 66.042 | 2.53 |  | mrMLM |
| CI | rs28447 | 2D | A/T | 81.616 | 2.65 | MLM | mrMLM |
| CI | rs30894 | 7B | G/A | 51.193 | 2.52 |  | mrMLM |
| CI | rs31181 | 5B | A/G | 26.242 | 2.61 |  | MLM |
| CI | rs31182 | 5B | G/C | 26.242 | 2.61 |  | MLM |
| CI | rs32494 | 3B | A/G | 65.99 | 2.58 |  | MLM |
| CI | rs32500 | 2D | A/G | 81.616 | 2.64 | MLM | mrMLM |
| CI | rs33218 | 3B | C/G | 54.619 | 2.63 | MLM | mrMLM |
| CI | rs34317 | 6A | C/G | 53.619 | 2.61 | MLM | mrMLM |
| CI | rs34865 | 2B | A/G | 111.506 | 2.48 |  | mrMLM |
| CI | rs34866 | 2B | A/C | 111.506 | 2.48 |  | mrMLM |
| CI | rs36493 | 5A | C/T | 10.23 | 2.61 |  | MLM |
| CI | rs36494 | 5A | C/T | 10.23 | 2.61 |  | MLM |
| CI | rs37116 | 6B | C/G | 43.284 | 2.70 | MLM | mrMLM |
| CI | rs3724 | 1B | C/T | 32.984 | 2.58 |  | MLM |
| CI | rs39629 | 3A | G/T | 112.886 | 2.75 | MLM | mrMLM |
| CI | rs39630 | 3A | T/C | 112.886 | 2.71 | MLM | mrMLM |
| CI | rs39631 | 3A | C/G | 112.886 | 2.75 | MLM | mrMLM |
| CI | rs44265 | 3B | A/G | 54.619 | 2.57 |  | mrMLM |
| CI | rs46204 | 1B | C/T | 70.6 | 2.59 | MLM | mrMLM |
| CI | rs46898 | 6A | A/T | 99.391 | 2.58 |  | MLM |
| CI | rs48837 | 3A | A/G | 37.753 | 2.52 |  | mrMLM |
| CI | rs50039 | 3B | C/T | 54.619 | 2.58 |  | MLM |
| CI | rs50947 | 5B | A/T | 18.219 | 2.58 |  | MLM |
| CI | rs5126 | 7B | C/T | 51.193 | 2.54 |  | mrMLM |
| CI | rs5185 | 2D | A/C | 81.616 | 2.62 |  | MLM |
| CI | rs54172 | 3B | A/G | 56.892 | 2.45 |  | mrMLM |
| CI | rs56301 | UN | C/T | 0 | 2.58 |  | MLM |
| CI | rs57076 | 2D | C/G | 81.616 | 2.59 | MLM | mrMLM |
| CI | rs57124 | 6B | A/G | 92.187 | 2.54 |  | mrMLM |
| CI | rs57400 | 5B | A/G | 26.242 | 2.72 | MLM | mrMLM |
| CI | rs5800 | 2B | T/C | 77.383 | 2.60 | MLM | mrMLM |
| CI | rs58019 | 2A | A/G | 87.971 | 2.41 |  | mrMLM |
| CI | rs58765 | 2B | A/G | 60.32 | 2.69 | MLM | mrMLM |
| CI | rs59033 | 4A | A/C | 62.152 | 2.33 |  | mrMLM |
| CI | rs60745 | 3B | A/G | 54.619 | 2.57 |  | mrMLM |
| CI | rs60746 | 3B | A/G | 54.619 | 2.61 | MLM | mrMLM |
| CI | rs61560 | 1B | A/G | 47.847 | 2.46 |  | mrMLM |
| CI | rs6166 | 2A | C/G | 74.319 | 2.54 |  | mrMLM |
| CI | rs63205 | 5A | C/T | 10.23 | 2.59 |  | MLM |
| CI | rs63206 | 5A | T/C | 10.23 | 2.59 |  | MLM |
| CI | rs6456 | 2B | C/G | 60.32 | 2.61 | MLM | mrMLM |
| CI | rs6872 | 5B | C/T | 127.712 | 2.51 |  | mrMLM |
| CI | rs9286 | 6A | C/T | 99.391 | 2.60 |  | MLM |
| CI | rs9410 | 3B | A/G | 40.978 | 2.62 |  | MLM |
| CI | rs9504 | 3A | A/G | 10.254 | 2.57 |  | mrMLM |
| FDS | rs12012 | 2A | A/G | 74.319 | 2.52 |  | mrMLM |
| FDS | rs13733 | 2A | C/T | 10.254 | 2.58 | MLM | mrMLM |
| FDS | rs13744 | 6A | A/G | 99.391 | 2.59 |  | MLM |
| FDS | rs13923 | 5A | C/T | 10.23 | 2.58 |  | MLM |
| FDS | rs13924 | 5A | C/G | 10.23 | 2.57 |  | MLM |
| FDS | rs15134 | 2A | C/T | 59.228 | 2.52 |  | mrMLM |
| FDS | rs17183 | 7B | C/T | 118.551 | 2.44 |  | mrMLM |
| FDS | rs17332 | 1B | A/G | 88.795 | 2.47 |  | mrMLM |
| FDS | rs18379 | 1B | C/T | 75.148 | 2.59 | MLM | mrMLM |
| FDS | rs18695 | 6D | A/G | 55.761 | 2.81 | MLM | mrMLM |
| FDS | rs20790 | 3B | A/G | 55.755 | 2.66 | MLM | mrMLM |
| FDS | rs20851 | 6B | C/T | 47.263 | 2.54 | MLM | mrMLM |
| FDS | rs21979 | 1B | C/T | 113.814 | 2.63 | MLM | mrMLM |
| FDS | rs23749 | 7B | A/G | 115.14 | 2.57 | MLM | mrMLM |
| FDS | rs2383 | 2A | C/T | 74.319 | 2.69 | MLM | mrMLM |
| FDS | rs2385 | 2D | A/G | 71.387 | 2.47 |  | mrMLM |
| FDS | rs2386 | 2D | C/G | 71.387 | 2.47 |  | mrMLM |
| FDS | rs23886 | 6B | C/T | 43.284 | 2.56 |  | MLM |
| FDS | rs24174 | 3B | A/T | 54.619 | 2.60 | MLM | mrMLM |
| FDS | rs24434 | 3B | C/T | 54.619 | 2.56 |  | MLM |
| FDS | rs24786 | 1B | A/G | 66.042 | 2.47 |  | mrMLM |
| FDS | rs24788 | 1B | G/A | 66.042 | 2.49 |  | mrMLM |
| FDS | rs25504 | 5D | C/T | 33.541 | 2.63 | MLM | mrMLM |
| FDS | rs26028 | 3B | C/T | 55.755 | 2.65 | MLM | mrMLM |
| FDS | rs26220 | 1B | G/T | 66.042 | 2.76 | MLM | mrMLM |
| FDS | rs26318 | 2D | C/G | 81.616 | 2.80 | MLM | mrMLM |
| FDS | rs26889 | 2B | A/T | 60.32 | 2.78 | MLM | mrMLM |
| FDS | rs2720 | 2D | A/C | 81.616 | 2.59 |  | MLM |
| FDS | rs28342 | 1B | G/T | 66.042 | 2.47 |  | mrMLM |
| FDS | rs28343 | 1B | C/T | 66.042 | 2.47 |  | mrMLM |
| FDS | rs28447 | 2D | A/T | 81.616 | 2.63 |  | MLM |
| FDS | rs30894 | 7B | G/A | 51.193 | 2.46 |  | mrMLM |
| FDS | rs31181 | 5B | A/G | 26.242 | 2.59 |  | MLM |
| FDS | rs31182 | 5B | G/C | 26.242 | 2.59 |  | MLM |
| FDS | rs32500 | 2D | A/G | 81.616 | 2.62 |  | MLM |
| FDS | rs33218 | 3B | C/G | 54.619 | 2.61 | MLM | mrMLM |
| FDS | rs34317 | 6A | C/G | 53.619 | 2.63 | MLM | mrMLM |
| FDS | rs34865 | 2B | A/G | 111.506 | 2.46 |  | mrMLM |
| FDS | rs34866 | 2B | A/C | 111.506 | 2.46 |  | mrMLM |
| FDS | rs36174 | 6B | C/T | 94.461 | 2.55 |  | mrMLM |
| FDS | rs36493 | 5A | C/T | 10.23 | 2.62 |  | MLM |
| FDS | rs36494 | 5A | C/T | 10.23 | 2.62 |  | MLM |
| FDS | rs37116 | 6B | C/G | 43.284 | 2.59 | MLM | mrMLM |
| FDS | rs39629 | 3A | G/T | 112.886 | 2.78 | MLM | mrMLM |
| FDS | rs39630 | 3A | T/C | 112.886 | 2.75 | MLM | mrMLM |
| FDS | rs39631 | 3A | C/G | 112.886 | 2.78 | MLM | mrMLM |
| FDS | rs44265 | 3B | A/G | 54.619 | 2.56 | MLM | mrMLM |
| FDS | rs46204 | 1B | C/T | 70.6 | 2.56 | MLM | mrMLM |
| FDS | rs46898 | 6A | A/T | 99.391 | 2.61 |  | MLM |
| FDS | rs4840 | 7B | A/C | 51.193 | 2.58 | MLM | mrMLM |
| FDS | rs48837 | 3A | A/G | 37.753 | 2.49 |  | mrMLM |
| FDS | rs50039 | 3B | C/T | 54.619 | 2.57 |  | MLM |
| FDS | rs50947 | 5B | A/T | 18.219 | 2.58 |  | MLM |
| FDS | rs50982 | 6A | C/G | 99.391 | 2.60 |  | MLM |
| FDS | rs5126 | 7B | C/T | 51.193 | 2.48 |  | mrMLM |
| FDS | rs5185 | 2D | A/C | 81.616 | 2.60 |  | MLM |
| FDS | rs54172 | 3B | A/G | 56.892 | 2.41 |  | mrMLM |
| FDS | rs56301 | UN | C/T | 0 | 2.57 |  | MLM |
| FDS | rs57076 | 2D | C/G | 81.616 | 2.57 |  | MLM |
| FDS | rs57400 | 5B | A/G | 26.242 | 2.74 | MLM | mrMLM |
| FDS | rs5800 | 2B | T/C | 77.383 | 2.58 | MLM | mrMLM |
| FDS | rs58019 | 2A | A/G | 87.971 | 2.43 |  | mrMLM |
| FDS | rs58765 | 2B | A/G | 60.32 | 2.64 | MLM | mrMLM |
| FDS | rs59033 | 4A | A/C | 62.152 | 2.32 |  | mrMLM |
| FDS | rs5936 | 1A | A/G | 68.388 | 2.58 |  | MLM |
| FDS | rs59523 | 1A | C/T | 3.415 | 2.54 | MLM | mrMLM |
| FDS | rs59987 | 1B | A/C | 36.398 | 2.57 |  | MLM |
| FDS | rs60745 | 3B | A/G | 54.619 | 2.55 |  | mrMLM |
| FDS | rs60746 | 3B | A/G | 54.619 | 2.59 | MLM | mrMLM |
| FDS | rs61560 | 1B | A/G | 47.847 | 2.44 |  | mrMLM |
| FDS | rs6166 | 2A | C/G | 74.319 | 2.49 |  | mrMLM |
| FDS | rs63205 | 5A | C/T | 10.23 | 2.59 |  | MLM |
| FDS | rs63206 | 5A | T/C | 10.23 | 2.59 |  | MLM |
| FDS | rs6456 | 2B | C/G | 60.32 | 2.54 |  | mrMLM |
| FDS | rs6872 | 5B | C/T | 127.712 | 2.47 |  | mrMLM |
| FDS | rs784 | 4A | C/G | 9.109 | 2.57 |  | MLM |
| FDS | rs8636 | 4A | G/T | 62.152 | 2.35 |  | mrMLM |
| FDS | rs9286 | 6A | C/T | 99.391 | 2.66 |  | MLM |
| FDS | rs9410 | 3B | A/G | 40.978 | 2.57 |  | MLM |
| FDS | rs9504 | 3A | A/G | 10.254 | 2.55 |  | mrMLM |
| LR_97_12 | rs10259 | 2B | C/T | 0 | 2.37 |  | mrMLM |
| LR_97_12 | rs11920 | 7A | A/G | 63.946 | 2.44 | MLM | mrMLM |
| LR_97_12 | rs13728 | 7A | A/G | 63.946 | 2.61 | MLM | mrMLM |
| LR_97_12 | rs14625 | 7A | A/G | 123.112 | 2.38 |  | mrMLM |
| LR_97_12 | rs1547 | UN | A/T | 0 | 2.39 | MLM | mrMLM |
| LR_97_12 | rs15875 | 6B | C/A | 85.356 | 2.50 | MLM | mrMLM |
| LR_97_12 | rs15876 | 6B | A/C | 85.356 | 2.49 | MLM | mrMLM |
| LR_97_12 | rs16481 | 2D | A/T | 13.642 | 2.46 | MLM | mrMLM |
| LR_97_12 | rs16781 | 7A | C/G | 110.567 | 2.46 | MLM | mrMLM |
| LR_97_12 | rs16782 | 7A | T/C | 110.567 | 2.46 | MLM | mrMLM |
| LR_97_12 | rs1798 | 5D | A/G | 83.13 | 2.46 | MLM | mrMLM |
| LR_97_12 | rs1799 | 5D | A/G | 83.13 | 2.46 | MLM | mrMLM |
| LR_97_12 | rs18979 | 3B | G/T | 48.935 | 2.40 |  | MLM |
| LR_97_12 | rs1919 | 2B | A/G | 96.718 | 2.39 |  | MLM |
| LR_97_12 | rs19912 | 5B | G/T | 39.337 | 2.42 | MLM | mrMLM |
| LR_97_12 | rs2007 | 4A | G/A | 124.821 | 2.48 | MLM | mrMLM |
| LR_97_12 | rs2008 | 4A | C/T | 124.821 | 2.48 | MLM | mrMLM |
| LR_97_12 | rs2021 | 3B | T/C | 60.303 | 2.42 |  | MLM |
| LR_97_12 | rs20602 | 6B | C/T | 85.356 | 2.42 | MLM | mrMLM |
| LR_97_12 | rs20688 | 5D | A/G | 83.13 | 2.38 |  | mrMLM |
| LR_97_12 | rs20689 | 5D | T/C | 83.13 | 2.38 |  | mrMLM |
| LR_97_12 | rs20781 | 4B | A/G | 57.77 | 2.40 | MLM | mrMLM |
| LR_97_12 | rs20782 | 4B | A/T | 57.77 | 2.47 | MLM | mrMLM |
| LR_97_12 | rs21489 | UN | A/G | 0 | 2.25 |  | mrMLM |
| LR_97_12 | rs22923 | 4A | A/G | 61.015 | 2.21 |  | mrMLM |
| LR_97_12 | rs2359 | 4A | A/C | 136.758 | 2.41 | MLM | mrMLM |
| LR_97_12 | rs2360 | 4A | A/C | 136.758 | 2.41 | MLM | mrMLM |
| LR_97_12 | rs2361 | 4A | C/T | 136.758 | 2.41 | MLM | mrMLM |
| LR_97_12 | rs23684 | 2A | A/G | 11.39 | 2.42 |  | MLM |
| LR_97_12 | rs24374 | 6B | C/T | 58.062 | 2.54 | MLM | mrMLM |
| LR_97_12 | rs24375 | 6B | T/C | 58.062 | 2.54 | MLM | mrMLM |
| LR_97_12 | rs24376 | 6B | T/C | 58.062 | 2.54 | MLM | mrMLM |
| LR_97_12 | rs2448 | 1B | A/G | 47.847 | 2.32 |  | mrMLM |
| LR_97_12 | rs2483 | 2B | A/G | 0 | 2.41 |  | MLM |
| LR_97_12 | rs25744 | 5B | A/G | 11.394 | 2.42 | MLM | mrMLM |
| LR_97_12 | rs26013 | UN | G/A | 0 | 2.22 |  | mrMLM |
| LR_97_12 | rs27896 | 5D | A/C | 36.382 | 2.42 |  | MLM |
| LR_97_12 | rs27897 | 5D | C/A | 36.382 | 2.42 |  | MLM |
| LR_97_12 | rs2800 | 4A | C/G | 136.19 | 2.40 | MLM | mrMLM |
| LR_97_12 | rs2808 | 6D | A/T | 115.958 | 2.33 |  | mrMLM |
| LR_97_12 | rs28647 | 6B | A/G | 63.746 | 2.45 | MLM | mrMLM |
| LR_97_12 | rs28795 | 2D | C/G | 45.24 | 2.43 | MLM | mrMLM |
| LR_97_12 | rs30183 | 2B | A/G | 72.825 | 2.37 |  | mrMLM |
| LR_97_12 | rs30955 | 3A | C/T | 54.806 | 2.40 |  | MLM |
| LR_97_12 | rs31671 | 2D | G/T | 13.642 | 2.44 | MLM | mrMLM |
| LR_97_12 | rs32284 | 1B | A/G | 91.068 | 2.57 | MLM | mrMLM |
| LR_97_12 | rs32285 | 1B | A/G | 91.068 | 2.57 | MLM | mrMLM |
| LR_97_12 | rs33082 | 7B | A/G | 93.505 | 2.15 |  | mrMLM |
| LR_97_12 | rs33083 | 7B | G/A | 93.505 | 2.15 |  | mrMLM |
| LR_97_12 | rs33837 | 4B | A/G | 60.612 | 2.40 |  | MLM |
| LR_97_12 | rs34096 | 2D | A/G | 13.642 | 2.36 |  | mrMLM |
| LR_97_12 | rs3411 | UN | A/C | 0 | 2.51 | MLM | mrMLM |
| LR_97_12 | rs34816 | 5B | A/G | 38.769 | 2.50 | MLM | mrMLM |
| LR_97_12 | rs34817 | 5B | G/A | 38.769 | 2.50 | MLM | mrMLM |
| LR_97_12 | rs35462 | 7B | A/G | 93.505 | 2.13 |  | mrMLM |
| LR_97_12 | rs36959 | 2B | C/T | 60.32 | 2.45 |  | MLM |
| LR_97_12 | rs37304 | 1B | C/T | 44.438 | 2.22 |  | mrMLM |
| LR_97_12 | rs37679 | 2B | A/C | 60.32 | 2.42 |  | MLM |
| LR_97_12 | rs3778 | 6B | A/G | 63.178 | 2.43 |  | MLM |
| LR_97_12 | rs38255 | 3B | A/G | 113.379 | 2.43 | MLM | mrMLM |
| LR_97_12 | rs38397 | 7B | G/A | 55.744 | 2.44 | MLM | mrMLM |
| LR_97_12 | rs39305 | 7B | C/T | 43.237 | 2.41 |  | MLM |
| LR_97_12 | rs39491 | UN | A/G | 0 | 2.35 |  | mrMLM |
| LR_97_12 | rs39527 | 4A | A/G | 61.015 | 2.41 |  | MLM |
| LR_97_12 | rs39680 | 2B | G/T | 66.573 | 2.46 | MLM | mrMLM |
| LR_97_12 | rs41570 | 6B | C/T | 62.609 | 2.69 | MLM | mrMLM |
| LR_97_12 | rs41571 | 6B | G/C | 62.609 | 2.69 | MLM | mrMLM |
| LR_97_12 | rs42166 | 7B | A/C | 43.237 | 2.46 | MLM | mrMLM |
| LR_97_12 | rs43378 | 5A | C/T | 9.093 | 2.40 |  | MLM |
| LR_97_12 | rs43827 | 5A | A/G | 82.286 | 2.35 |  | mrMLM |
| LR_97_12 | rs43828 | 5A | T/C | 82.286 | 2.35 |  | mrMLM |
| LR_97_12 | rs43924 | 6B | C/T | 62.609 | 2.47 | MLM | mrMLM |
| LR_97_12 | rs440 | 5D | A/G | 83.13 | 2.36 |  | mrMLM |
| LR_97_12 | rs441 | 5D | G/A | 83.13 | 2.36 |  | mrMLM |
| LR_97_12 | rs45130 | 7B | A/G | 69.39 | 2.44 | MLM | mrMLM |
| LR_97_12 | rs45131 | 7B | C/T | 69.39 | 2.43 | MLM | mrMLM |
| LR_97_12 | rs45802 | 7B | A/C | 92.919 | 2.17 |  | mrMLM |
| LR_97_12 | rs4602 | 5B | C/T | 35.928 | 2.56 | MLM | mrMLM |
| LR_97_12 | rs46046 | 3B | G/A | 45.525 | 2.40 | MLM | mrMLM |
| LR_97_12 | rs46842 | 3B | C/G | 45.525 | 2.39 |  | MLM |
| LR_97_12 | rs46843 | 3B | C/A | 45.525 | 2.39 |  | MLM |
| LR_97_12 | rs47722 | 7B | C/T | 92.332 | 2.40 |  | mrMLM |
| LR_97_12 | rs48221 | 6A | C/G | 68.585 | 2.44 |  | MLM |
| LR_97_12 | rs48269 | 6B | C/G | 47.831 | 2.28 |  | mrMLM |
| LR_97_12 | rs48776 | 5B | A/C | 62.719 | 2.58 | MLM | mrMLM |
| LR_97_12 | rs4961 | 6B | A/G | 62.609 | 2.43 | MLM | mrMLM |
| LR_97_12 | rs50047 | 3B | A/C | 22.764 | 2.37 |  | mrMLM |
| LR_97_12 | rs51004 | 2B | A/G | 66.004 | 2.58 | MLM | mrMLM |
| LR_97_12 | rs51217 | 6B | C/T | 85.356 | 2.39 | MLM | mrMLM |
| LR_97_12 | rs51220 | 6B | C/T | 85.356 | 2.39 | MLM | mrMLM |
| LR_97_12 | rs51367 | 6B | A/T | 46.694 | 2.55 | MLM | mrMLM |
| LR_97_12 | rs5269 | UN | C/G | 0 | 2.28 |  | mrMLM |
| LR_97_12 | rs53036 | 2B | A/G | 72.825 | 2.52 | MLM | mrMLM |
| LR_97_12 | rs53835 | 4A | C/T | 111.179 | 2.26 |  | mrMLM |
| LR_97_12 | rs55703 | 6B | A/T | 85.356 | 2.43 | MLM | mrMLM |
| LR_97_12 | rs55718 | 5A | C/T | 87.98 | 2.38 |  | mrMLM |
| LR_97_12 | rs56069 | 7B | A/C | 112.867 | 2.40 |  | MLM |
| LR_97_12 | rs56070 | 7B | C/A | 112.867 | 2.40 |  | MLM |
| LR_97_12 | rs57719 | 6B | C/T | 85.356 | 2.43 | MLM | mrMLM |
| LR_97_12 | rs57797 | 6B | A/G | 62.609 | 2.53 | MLM | mrMLM |
| LR_97_12 | rs58203 | 5D | G/T | 83.13 | 2.36 |  | mrMLM |
| LR_97_12 | rs58632 | 1B | A/G | 46.143 | 2.45 | MLM | mrMLM |
| LR_97_12 | rs59326 | 6A | A/G | 94.843 | 2.38 |  | mrMLM |
| LR_97_12 | rs59356 | 4A | A/T | 136.19 | 2.40 | MLM | mrMLM |
| LR_97_12 | rs59894 | 6D | A/G | 54.625 | 2.40 |  | MLM |
| LR_97_12 | rs60947 | 3A | A/G | 23.905 | 2.53 | MLM | mrMLM |
| LR_97_12 | rs6607 | 7A | C/T | 4.551 | 2.40 | MLM | mrMLM |
| LR_97_12 | rs7934 | 3A | C/T | 9.116 | 2.52 | MLM | mrMLM |
| LR_97_12 | rs8904 | 7B | A/G | 94.66 | 2.41 |  | MLM |
| LR_97_12 | rs9211 | 1B | C/T | 46.711 | 2.67 | MLM | mrMLM |
| LR_97_12 | rs9212 | 1B | C/T | 46.711 | 2.74 | MLM | mrMLM |
| LR_97_12 | rs9233 | 5B | A/G | 62.719 | 2.39 | MLM | mrMLM |
| LR_98_12 | rs10220 | 3D | A/G | 38.873 | 1.97 |  | mrMLM |
| LR_98_12 | rs10978 | 5A | G/T | 47.99 | 1.95 |  | mrMLM |
| LR_98_12 | rs11268 | 7B | G/A | 114.004 | 2.03 |  | mrMLM |
| LR_98_12 | rs11415 | 7B | A/G | 51.193 | 2.01 |  | mrMLM |
| LR_98_12 | rs11752 | 7B | A/G | 114.004 | 2.03 |  | mrMLM |
| LR_98_12 | rs12518 | 7B | A/G | 50.057 | 2.05 | MLM | mrMLM |
| LR_98_12 | rs13328 | 3D | A/G | 38.873 | 1.99 |  | mrMLM |
| LR_98_12 | rs13441 | 3D | C/T | 38.873 | 1.97 | MLM | mrMLM |
| LR_98_12 | rs14417 | 3D | A/T | 41.146 | 2.02 |  | mrMLM |
| LR_98_12 | rs14922 | 2B | A/G | 59.184 | 2.00 |  | mrMLM |
| LR_98_12 | rs15492 | 4A | A/G | 121.41 | 2.09 | MLM | mrMLM |
| LR_98_12 | rs1653 | 3B | C/T | 39.841 | 1.96 |  | mrMLM |
| LR_98_12 | rs18054 | 7A | A/G | 65.083 | 2.13 | MLM | mrMLM |
| LR_98_12 | rs184 | 6D | A/G | 39.846 | 2.01 |  | mrMLM |
| LR_98_12 | rs20688 | 5D | A/G | 83.13 | 2.00 |  | mrMLM |
| LR_98_12 | rs20689 | 5D | T/C | 83.13 | 2.00 |  | mrMLM |
| LR_98_12 | rs20781 | 4B | A/G | 57.77 | 2.08 | MLM | mrMLM |
| LR_98_12 | rs20782 | 4B | A/T | 57.77 | 2.09 | MLM | mrMLM |
| LR_98_12 | rs23749 | 7B | A/G | 115.14 | 2.11 | MLM | mrMLM |
| LR_98_12 | rs25737 | 3B | C/T | 45.525 | 2.23 | MLM | mrMLM |
| LR_98_12 | rs27584 | 1A | C/T | 32.003 | 1.92 |  | mrMLM |
| LR_98_12 | rs28795 | 2D | C/G | 45.24 | 1.93 | MLM | mrMLM |
| LR_98_12 | rs30183 | 2B | A/G | 72.825 | 2.00 |  | mrMLM |
| LR_98_12 | rs30371 | 7B | C/T | 114.004 | 2.02 |  | mrMLM |
| LR_98_12 | rs31061 | 7D | G/T | 7.962 | 2.04 |  | mrMLM |
| LR_98_12 | rs33422 | 7B | C/G | 109.456 | 1.95 |  | mrMLM |
| LR_98_12 | rs33423 | 7B | C/G | 109.456 | 1.95 |  | mrMLM |
| LR_98_12 | rs33491 | 2B | A/T | 59.184 | 1.99 |  | mrMLM |
| LR_98_12 | rs34114 | 3D | G/T | 38.873 | 1.96 |  | mrMLM |
| LR_98_12 | rs34115 | 3D | A/G | 38.873 | 1.96 |  | mrMLM |
| LR_98_12 | rs34241 | 3B | C/T | 45.525 | 2.03 |  | mrMLM |
| LR_98_12 | rs34242 | 3B | A/G | 45.525 | 2.05 | MLM | mrMLM |
| LR_98_12 | rs34916 | 3D | C/T | 53.673 | 1.98 |  | mrMLM |
| LR_98_12 | rs34968 | 7B | A/C | 114.004 | 2.05 | MLM | mrMLM |
| LR_98_12 | rs35918 | 7A | A/C | 109.43 | 2.04 |  | mrMLM |
| LR_98_12 | rs36084 | 5B | C/T | 45.594 | 2.13 | MLM | mrMLM |
| LR_98_12 | rs36215 | 1B | A/T | 45.006 | 1.88 |  | mrMLM |
| LR_98_12 | rs36483 | 6B | G/T | 94.461 | 2.22 | MLM | mrMLM |
| LR_98_12 | rs36484 | 6B | C/G | 94.461 | 2.22 | MLM | mrMLM |
| LR_98_12 | rs36491 | 2D | C/T | 54.335 | 2.02 |  | mrMLM |
| LR_98_12 | rs36555 | 6B | G/A | 47.831 | 2.20 | MLM | mrMLM |
| LR_98_12 | rs36886 | 5B | A/G | 26.242 | 2.01 | MLM | mrMLM |
| LR_98_12 | rs38875 | 5B | C/T | 133.397 | 2.08 |  | MLM |
| LR_98_12 | rs39305 | 7B | C/T | 43.237 | 2.05 |  | MLM |
| LR_98_12 | rs39944 | 2B | A/G | 59.184 | 1.97 |  | mrMLM |
| LR_98_12 | rs40554 | 5A | A/C | 9.093 | 1.97 |  | mrMLM |
| LR_98_12 | rs40555 | 5A | C/T | 9.093 | 1.97 |  | mrMLM |
| LR_98_12 | rs40556 | 5A | G/A | 9.093 | 1.97 |  | mrMLM |
| LR_98_12 | rs40575 | 3B | A/G | 48.935 | 1.91 |  | mrMLM |
| LR_98_12 | rs41991 | 2B | A/G | 59.184 | 2.03 |  | mrMLM |
| LR_98_12 | rs41993 | 2B | A/G | 59.184 | 2.01 |  | mrMLM |
| LR_98_12 | rs42326 | 2A | A/G | 92.517 | 1.93 |  | mrMLM |
| LR_98_12 | rs440 | 5D | A/G | 83.13 | 2.01 |  | mrMLM |
| LR_98_12 | rs441 | 5D | G/A | 83.13 | 2.01 |  | mrMLM |
| LR_98_12 | rs44885 | 5D | C/T | 7.959 | 1.97 |  | mrMLM |
| LR_98_12 | rs44886 | 5D | A/G | 7.959 | 1.97 |  | mrMLM |
| LR_98_12 | rs45413 | 6D | A/G | 119.937 | 2.05 |  | MLM |
| LR_98_12 | rs45993 | 5A | A/G | 90.253 | 2.04 |  | mrMLM |
| LR_98_12 | rs46728 | 7D | C/T | 7.962 | 2.07 | MLM | mrMLM |
| LR_98_12 | rs49715 | 5D | A/G | 168.429 | 2.04 |  | mrMLM |
| LR_98_12 | rs50047 | 3B | A/C | 22.764 | 2.24 | MLM | mrMLM |
| LR_98_12 | rs50765 | 5B | C/T | 80.923 | 1.98 |  | mrMLM |
| LR_98_12 | rs51251 | 1B | A/G | 111.539 | 2.05 |  | MLM |
| LR_98_12 | rs51542 | 6D | C/T | 48.941 | 2.01 |  | mrMLM |
| LR_98_12 | rs52133 | 5A | C/T | 9.093 | 1.93 |  | mrMLM |
| LR_98_12 | rs52134 | 5A | G/C | 9.093 | 1.93 |  | mrMLM |
| LR_98_12 | rs52542 | 7B | C/G | 114.004 | 2.09 | MLM | mrMLM |
| LR_98_12 | rs57916 | 5B | G/T | 92.298 | 2.01 |  | mrMLM |
| LR_98_12 | rs58203 | 5D | G/T | 83.13 | 2.03 |  | mrMLM |
| LR_98_12 | rs58692 | 4D | C/T | 90.119 | 2.09 |  | MLM |
| LR_98_12 | rs58952 | 5D | G/T | 83.13 | 2.08 | MLM | mrMLM |
| LR_98_12 | rs59576 | 2D | A/G | 73.66 | 1.86 |  | mrMLM |
| LR_98_12 | rs60975 | 5A | C/G | 90.253 | 2.04 | MLM | mrMLM |
| LR_98_12 | rs6136 | UN | A/G | 0 | 2.10 | MLM | mrMLM |
| LR_98_12 | rs62731 | 2D | T/C | 11.368 | 1.97 |  | mrMLM |
| LR_98_12 | rs62732 | 2D | A/C | 11.368 | 1.97 |  | mrMLM |
| LR_98_12 | rs62733 | 2D | C/T | 11.368 | 1.97 |  | mrMLM |
| LR_98_12 | rs62734 | 2D | C/A | 11.368 | 1.97 |  | mrMLM |
| LR_98_12 | rs62871 | 3D | A/C | 38.873 | 1.97 |  | mrMLM |
| LR_98_12 | rs62902 | 6B | C/G | 47.831 | 2.07 | MLM | mrMLM |
| LR_98_12 | rs62903 | 6B | C/G | 47.831 | 2.06 | MLM | mrMLM |
| LR_98_12 | rs63851 | 5A | A/G | 111.398 | 2.02 |  | mrMLM |
| LR_98_12 | rs7342 | 7A | A/G | 108.293 | 2.00 |  | mrMLM |
| LR_98_12 | rs7934 | 3A | C/T | 9.116 | 2.08 | MLM | mrMLM |
| LR_98_12 | rs9493 | 3B | A/G | 22.764 | 2.04 |  | mrMLM |
| LR_98_22 | rs11410 | 6D | A/G | 52.35 | 2.87 | MLM | mrMLM |
| LR_98_22 | rs12115 | 7D | A/G | 143.803 | 2.89 | MLM | mrMLM |
| LR_98_22 | rs13076 | 5B | C/T | 39.337 | 2.97 | MLM | mrMLM |
| LR_98_22 | rs13077 | 5B | G/A | 39.337 | 2.97 | MLM | mrMLM |
| LR_98_22 | rs15134 | 2A | C/T | 59.228 | 2.87 |  | mrMLM |
| LR_98_22 | rs15492 | 4A | A/G | 121.41 | 2.90 | MLM | mrMLM |
| LR_98_22 | rs16835 | 5A | G/A | 103.987 | 2.91 | MLM | mrMLM |
| LR_98_22 | rs17035 | 1A | A/G | 3.415 | 2.88 | MLM | mrMLM |
| LR_98_22 | rs17210 | 2A | C/T | 92.517 | 2.92 | MLM | mrMLM |
| LR_98_22 | rs17332 | 1B | A/G | 88.795 | 2.89 |  | mrMLM |
| LR_98_22 | rs1798 | 5D | A/G | 83.13 | 2.96 | MLM | mrMLM |
| LR_98_22 | rs1799 | 5D | A/G | 83.13 | 2.96 | MLM | mrMLM |
| LR_98_22 | rs19397 | 2B | T/C | 72.825 | 2.87 |  | MLM |
| LR_98_22 | rs19912 | 5B | G/T | 39.337 | 2.91 | MLM | mrMLM |
| LR_98_22 | rs20688 | 5D | A/G | 83.13 | 2.85 |  | mrMLM |
| LR_98_22 | rs20689 | 5D | T/C | 83.13 | 2.85 |  | mrMLM |
| LR_98_22 | rs20782 | 4B | A/T | 57.77 | 2.87 | MLM | mrMLM |
| LR_98_22 | rs21304 | 5A | C/T | 0 | 2.85 |  | mrMLM |
| LR_98_22 | rs21305 | 5A | T/C | 0 | 2.85 |  | mrMLM |
| LR_98_22 | rs21939 | 5B | C/G | 132.26 | 2.89 | MLM | mrMLM |
| LR_98_22 | rs22627 | 6A | C/T | 99.391 | 2.97 | MLM | mrMLM |
| LR_98_22 | rs23002 | 1A | C/G | 54.745 | 2.91 |  | MLM |
| LR_98_22 | rs2720 | 2D | A/C | 81.616 | 2.88 | MLM | mrMLM |
| LR_98_22 | rs27584 | 1A | C/T | 32.003 | 2.83 |  | mrMLM |
| LR_98_22 | rs2787 | 5D | G/A | 66.057 | 2.90 | MLM | mrMLM |
| LR_98_22 | rs28795 | 2D | C/G | 45.24 | 2.92 |  | mrMLM |
| LR_98_22 | rs30786 | 1A | C/T | 55.314 | 2.89 | MLM | mrMLM |
| LR_98_22 | rs30787 | 1A | C/T | 55.314 | 2.89 | MLM | mrMLM |
| LR_98_22 | rs35177 | 2B | A/G | 67.141 | 2.87 |  | MLM |
| LR_98_22 | rs36084 | 5B | C/T | 45.594 | 2.86 |  | mrMLM |
| LR_98_22 | rs36483 | 6B | G/T | 94.461 | 3.15 | MLM | mrMLM |
| LR_98_22 | rs36484 | 6B | C/G | 94.461 | 3.15 | MLM | mrMLM |
| LR_98_22 | rs372 | 4A | C/T | 76.969 | 2.89 | MLM | mrMLM |
| LR_98_22 | rs37446 | 6A | A/G | 25.146 | 2.96 | MLM | mrMLM |
| LR_98_22 | rs38269 | 7D | A/G | 143.803 | 2.88 | MLM | mrMLM |
| LR_98_22 | rs38270 | 7D | A/G | 143.803 | 2.88 | MLM | mrMLM |
| LR_98_22 | rs38875 | 5B | C/T | 133.397 | 2.94 | MLM | mrMLM |
| LR_98_22 | rs39305 | 7B | C/T | 43.237 | 3.02 | MLM | mrMLM |
| LR_98_22 | rs40575 | 3B | A/G | 48.935 | 2.80 |  | mrMLM |
| LR_98_22 | rs41211 | 2B | A/G | 72.825 | 2.88 |  | MLM |
| LR_98_22 | rs43451 | 2A | A/G | 59.228 | 2.87 |  | MLM |
| LR_98_22 | rs43452 | 2A | T/C | 59.228 | 2.87 |  | MLM |
| LR_98_22 | rs440 | 5D | A/G | 83.13 | 2.84 |  | mrMLM |
| LR_98_22 | rs441 | 5D | G/A | 83.13 | 2.84 |  | mrMLM |
| LR_98_22 | rs44885 | 5D | C/T | 7.959 | 2.96 | MLM | mrMLM |
| LR_98_22 | rs44886 | 5D | A/G | 7.959 | 2.96 | MLM | mrMLM |
| LR_98_22 | rs49543 | 1B | C/T | 56.943 | 3.01 | MLM | mrMLM |
| LR_98_22 | rs50047 | 3B | A/C | 22.764 | 3.01 | MLM | mrMLM |
| LR_98_22 | rs50194 | 7B | A/C | 112.867 | 2.86 |  | MLM |
| LR_98_22 | rs51191 | 5A | A/G | 34.125 | 2.87 |  | MLM |
| LR_98_22 | rs51367 | 6B | A/T | 46.694 | 2.94 | MLM | mrMLM |
| LR_98_22 | rs51983 | 5A | A/C | 93.664 | 2.87 | MLM | mrMLM |
| LR_98_22 | rs52026 | 5A | A/T | 93.664 | 2.98 | MLM | mrMLM |
| LR_98_22 | rs52548 | 2B | C/G | 72.825 | 3.02 | MLM | mrMLM |
| LR_98_22 | rs53036 | 2B | A/G | 72.825 | 3.06 | MLM | mrMLM |
| LR_98_22 | rs55992 | 7D | A/C | 157.445 | 2.88 | MLM | mrMLM |
| LR_98_22 | rs58203 | 5D | G/T | 83.13 | 2.84 |  | mrMLM |
| LR_98_22 | rs59493 | 4B | A/G | 56.065 | 2.88 | MLM | mrMLM |
| LR_98_22 | rs59576 | 2D | A/G | 73.66 | 3.07 | MLM | mrMLM |
| LR_98_22 | rs62568 | 4A | C/T | 61.015 | 2.94 |  | MLM |
| LR_98_22 | rs62825 | 7A | A/G | 0.569 | 2.87 |  | MLM |
| LR_98_22 | rs6313 | 2A | A/G | 74.319 | 2.88 | MLM | mrMLM |
| LR_98_22 | rs6314 | 2A | A/T | 74.319 | 2.88 | MLM | mrMLM |
| LR_98_22 | rs64114 | 3A | A/G | 111.749 | 2.87 |  | MLM |
| LR_98_22 | rs64115 | 3A | T/C | 111.749 | 2.87 |  | MLM |
| LR_98_22 | rs6441 | 2A | A/G | 59.228 | 2.90 | MLM | mrMLM |
| LR_98_22 | rs7091 | 2B | C/T | 86.479 | 2.94 | MLM | mrMLM |
| LR_98_22 | rs756 | 4D | A/G | 54.756 | 2.91 | MLM | mrMLM |
| LR_98_22 | rs7934 | 3A | C/T | 9.116 | 2.98 | MLM | mrMLM |
| LR_98_22 | rs8470 | 7A | A/G | 66.22 | 2.87 | MLM | mrMLM |
| LR_99_2 | rs11272 | 1B | A/G | 45.574 | 2.02 |  | mrMLM |
| LR_99_2 | rs11637 | 1B | A/G | 45.574 | 2.07 |  | mrMLM |
| LR_99_2 | rs11638 | 1B | A/G | 45.574 | 2.07 |  | mrMLM |
| LR_99_2 | rs12954 | 5D | A/G | 111.553 | 2.23 | MLM | mrMLM |
| LR_99_2 | rs14658 | 4A | A/G | 76.969 | 2.31 | MLM | mrMLM |
| LR_99_2 | rs15009 | 7A | C/G | 0 | 2.20 |  | MLM |
| LR_99_2 | rs15675 | 2B | C/T | 6.832 | 2.24 | MLM | mrMLM |
| LR_99_2 | rs15676 | 2B | C/T | 6.832 | 2.24 | MLM | mrMLM |
| LR_99_2 | rs1728 | UN | C/T | 0 | 2.20 |  | MLM |
| LR_99_2 | rs19379 | 3B | C/T | 64.283 | 2.14 |  | mrMLM |
| LR_99_2 | rs19380 | 3B | C/T | 64.283 | 2.14 |  | mrMLM |
| LR_99_2 | rs22061 | 7B | C/T | 93.505 | 2.16 |  | mrMLM |
| LR_99_2 | rs22505 | 6B | G/T | 48.967 | 2.11 |  | mrMLM |
| LR_99_2 | rs23129 | 7B | C/G | 2.273 | 2.14 |  | mrMLM |
| LR_99_2 | rs24338 | 7A | A/G | 123.112 | 2.17 |  | mrMLM |
| LR_99_2 | rs25744 | 5B | A/G | 11.394 | 2.07 |  | mrMLM |
| LR_99_2 | rs26180 | 3A | C/T | 110.613 | 2.19 | MLM | mrMLM |
| LR_99_2 | rs2808 | 6D | A/T | 115.958 | 2.13 |  | mrMLM |
| LR_99_2 | rs28795 | 2D | C/G | 45.24 | 2.08 |  | mrMLM |
| LR_99_2 | rs30506 | 1A | A/G | 80.9 | 2.36 | MLM | mrMLM |
| LR_99_2 | rs33082 | 7B | A/G | 93.505 | 2.16 |  | mrMLM |
| LR_99_2 | rs33083 | 7B | G/A | 93.505 | 2.16 |  | mrMLM |
| LR_99_2 | rs33205 | 4A | C/T | 76.401 | 2.20 | MLM | mrMLM |
| LR_99_2 | rs35 | 2A | A/G | 59.228 | 2.26 |  | MLM |
| LR_99_2 | rs35481 | 3A | A/G | 9.116 | 2.24 | MLM | mrMLM |
| LR_99_2 | rs35482 | 3A | G/A | 9.116 | 2.24 | MLM | mrMLM |
| LR_99_2 | rs35483 | 3A | G/T | 9.116 | 2.24 | MLM | mrMLM |
| LR_99_2 | rs35806 | 5B | T/G | 38.769 | 2.10 |  | mrMLM |
| LR_99_2 | rs35807 | 5B | T/G | 38.769 | 2.10 |  | mrMLM |
| LR_99_2 | rs35808 | 5B | C/T | 38.769 | 2.10 |  | mrMLM |
| LR_99_2 | rs35810 | 6D | A/G | 48.941 | 2.12 |  | mrMLM |
| LR_99_2 | rs35918 | 7A | A/C | 109.43 | 2.07 |  | mrMLM |
| LR_99_2 | rs36 | 2A | T/C | 59.228 | 2.26 |  | MLM |
| LR_99_2 | rs36215 | 1B | A/T | 45.006 | 2.12 |  | mrMLM |
| LR_99_2 | rs36229 | 7B | C/G | 51.193 | 2.22 | MLM | mrMLM |
| LR_99_2 | rs36959 | 2B | C/T | 60.32 | 2.27 | MLM | mrMLM |
| LR_99_2 | rs36960 | 2B | C/T | 60.32 | 2.25 |  | MLM |
| LR_99_2 | rs36960 | 2B | C/T | 60.32 | 2.22 | MLM | mrMLM |
| LR_99_2 | rs372 | 4A | C/T | 76.969 | 2.29 | MLM | mrMLM |
| LR_99_2 | rs38255 | 3B | A/G | 113.379 | 2.25 | MLM | mrMLM |
| LR_99_2 | rs38875 | 5B | C/T | 133.397 | 2.18 | MLM | mrMLM |
| LR_99_2 | rs39239 | 2A | A/G | 21.63 | 2.16 |  | MLM |
| LR_99_2 | rs40818 | 6B | C/T | 48.399 | 2.06 |  | mrMLM |
| LR_99_2 | rs41848 | 6D | A/G | 48.941 | 2.17 |  | mrMLM |
| LR_99_2 | rs44015 | 2B | C/T | 59.184 | 2.15 |  | mrMLM |
| LR_99_2 | rs44096 | 4B | C/G | 71.984 | 2.28 |  | mrMLM |
| LR_99_2 | rs44267 | 1B | C/G | 43.301 | 2.08 | MLM | mrMLM |
| LR_99_2 | rs44700 | 6A | C/G | 99.391 | 2.08 |  | mrMLM |
| LR_99_2 | rs44701 | 6A | A/G | 99.391 | 2.10 |  | mrMLM |
| LR_99_2 | rs44885 | 5D | C/T | 7.959 | 2.10 |  | mrMLM |
| LR_99_2 | rs44886 | 5D | A/G | 7.959 | 2.20 |  | mrMLM |
| LR_99_2 | rs45212 | 2A | C/T | 21.63 | 2.18 |  | MLM |
| LR_99_2 | rs45340 | 3B | A/T | 113.948 | 2.29 |  | mrMLM |
| LR_99_2 | rs45341 | 3B | A/T | 113.948 | 2.23 | MLM | mrMLM |
| LR_99_2 | rs47218 | 5B | A/G | 80.923 | 2.15 | MLM | mrMLM |
| LR_99_2 | rs47722 | 7B | C/T | 92.332 | 2.26 | MLM | mrMLM |
| LR_99_2 | rs47929 | 4A | A/G | 76.969 | 2.18 | MLM | mrMLM |
| LR_99_2 | rs50741 | 7B | G/C | 51.762 | 2.18 |  | mrMLM |
| LR_99_2 | rs50742 | 7B | C/T | 51.762 | 2.16 |  | mrMLM |
| LR_99_2 | rs51322 | 1B | A/C | 32.984 | 2.16 |  | mrMLM |
| LR_99_2 | rs51323 | 1B | C/T | 32.984 | 2.21 |  | mrMLM |
| LR_99_2 | rs51367 | 6B | A/T | 46.694 | 2.16 | MLM | mrMLM |
| LR_99_2 | rs51542 | 6D | C/T | 48.941 | 2.08 |  | mrMLM |
| LR_99_2 | rs53323 | 7A | A/G | 108.293 | 2.26 |  | mrMLM |
| LR_99_2 | rs53897 | 6A | A/G | 94.843 | 2.19 | MLM | mrMLM |
| LR_99_2 | rs56484 | 2A | A/C | 59.228 | 2.19 |  | MLM |
| LR_99_2 | rs56485 | 2A | G/A | 59.228 | 2.17 |  | MLM |
| LR_99_2 | rs56710 | 2B | A/G | 59.184 | 2.19 |  | mrMLM |
| LR_99_2 | rs57475 | 1A | A/G | 80.9 | 2.25 |  | MLM |
| LR_99_2 | rs58692 | 4D | C/T | 90.119 | 2.21 |  | MLM |
| LR_99_2 | rs59576 | 2D | A/G | 73.66 | 2.26 | MLM | mrMLM |
| LR_99_2 | rs598 | 3B | C/T | 26.175 | 2.12 | MLM | mrMLM |
| LR_99_2 | rs6018 | 3B | A/C | 113.379 | 2.30 |  | mrMLM |
| LR_99_2 | rs60617 | 6B | C/T | 43.284 | 2.11 | MLM | mrMLM |
| LR_99_2 | rs62675 | 3D | C/T | 121.901 | 2.58 |  | mrMLM |
| LR_99_2 | rs62902 | 6B | C/G | 47.831 | 2.57 | MLM | mrMLM |
| LR_99_2 | rs62903 | 6B | C/G | 47.831 | 2.19 | MLM | mrMLM |
| LR_99_2 | rs63129 | UN | A/C | 0 | 2.26 | MLM | mrMLM |
| LR_99_2 | rs6313 | 2A | A/G | 74.319 | 2.19 | MLM | mrMLM |
| LR_99_2 | rs63130 | UN | T/C | 0 | 2.26 | MLM | mrMLM |
| LR_99_2 | rs6314 | 2A | A/T | 74.319 | 2.15 | MLM | mrMLM |
| LR_99_2 | rs63294 | 2A | G/T | 74.319 | 2.15 |  | mrMLM |
| LR_99_2 | rs63295 | 2A | C/T | 74.319 | 2.09 |  | mrMLM |
| LR_99_2 | rs63793 | 1B | A/G | 75.717 | 2.05 |  | mrMLM |
| LR_99_2 | rs63795 | 1B | A/G | 75.717 | 2.13 |  | mrMLM |
| LR_99_2 | rs63851 | 5A | A/G | 111.398 | 2.15 |  | mrMLM |
| LR_99_2 | rs63973 | 4B | C/G | 58.338 | 2.15 |  | mrMLM |
| LR_99_2 | rs63974 | 4B | T/C | 58.338 | 2.18 |  | mrMLM |
| LR_99_2 | rs6549 | 2A | A/G | 11.39 | 2.24 |  | mrMLM |
| LR_99_2 | rs750 | 2B | C/T | 67.141 | 2.26 | MLM | mrMLM |
| LR_99_2 | rs7934 | 3A | C/T | 9.116 | 2.62 | MLM | mrMLM |
| LR_99_2 | rs9493 | 3B | A/G | 22.764 | 2.65 | MLM | mrMLM |

CHR: Chromosome

R^2^_:_ Coefficient of Determination

**Table S10**: Candidate genes around the reliable MTAs and their functional annotation for seedling and adult plant resistance.

| Traitname | SNP | CHR | Overlapping gene | Position | Biological process | Molecular function |
| --- | --- | --- | --- | --- | --- | --- |
| LR_98_22 | rs17035 | 1A | [TraesCS1A02G009000](https://plants.ensembl.org/Triticum_aestivum/Gene/Summary?db=core;g=TraesCS1A02G009000;tl=7Ww4HO1DFSSlH0HF-21600598-2334120922) | [5491167-5491230](https://plants.ensembl.org/Triticum_aestivum/Location/View?r=1A:5491163-5491233;tl=7Ww4HO1DFSSlH0HF-21600598-2334120922) |  | protein binding |
| LR_99_2 | rs30506 | 1A | [TraesCS1A02G031900](http://plants.ensembl.org/Triticum_aestivum/Gene/Summary?db=core;g=TraesCS1A02G031900;tl=q3Kh0T9JDxos5Qcr-21600637-2334130980) | [14610472-14610497](http://plants.ensembl.org/Triticum_aestivum/Location/View?r=1A:14610470-14610498;tl=q3Kh0T9JDxos5Qcr-21600637-2334130980) | protein phosphorylation | protein kinase activity, protein binding, ATP binding |
| LR_98_22 | rs23002 | 1A | TraesCS1A02G225000 | [395,172,063-395,180,663](http://plants.ensembl.org/Triticum_aestivum/Location/View?db=core;g=TraesCS1A02G225000;r=1A:395172063-395180663;tl=w7GBhUA5I1qEXKJ8-21592944-2333111792) | purine ribonucleoside monophosphate biosynthetic process, IMP salvage | AMP deaminase activity, deaminase activity |
| LR_97_12 | rs37304 | 1B | [TraesCS1B02G008600](http://plants.ensembl.org/Triticum_aestivum/Gene/Summary?db=core;g=TraesCS1B02G008600;tl=iHjZi4TBKRIemlUU-21600756-2334158872) | [4520136-4520155](http://plants.ensembl.org/Triticum_aestivum/Location/View?r=1B:4520135-4520155;tl=iHjZi4TBKRIemlUU-21600756-2334158872) |  | protein binding |
| LR_99_2 | rs11637 | 1B | [TraesCS1B02G243600](https://plants.ensembl.org/Triticum_aestivum/Gene/Summary?db=core;g=TraesCS1B02G243600;tl=y4vwUXp8q7a5j3ZF-21600245-2334055288) | [432560018-432560060](https://plants.ensembl.org/Triticum_aestivum/Location/View?r=1B:432560015-432560062;tl=y4vwUXp8q7a5j3ZF-21600245-2334055288) |  |  |
| AUDPC | rs3724 | 1B | TraesCS1B02G286200 | [497,799,346-497,822,159](http://plants.ensembl.org/Triticum_aestivum/Location/View?db=core;g=TraesCS1B02G286200;r=1B:497799346-497822159;t=TraesCS1B02G286200.1;tl=WXnFacBUYZeZAPzk-21592873-2333095756) | transmembrane transporter activity | transmembrane transport |
| LR_97_12 | rs2448 | 1B | [TraesCS1B02G299500](http://plants.ensembl.org/Triticum_aestivum/Gene/Summary?db=core;g=TraesCS1B02G299500;tl=Vmar1yCccMzkV0ck-21600195-2334049057) | [520470898-520470961](http://plants.ensembl.org/Triticum_aestivum/Location/View?r=1B:520470894-520470964;tl=Vmar1yCccMzkV0ck-21600195-2334049057) | vacuolar proton-transporting V-type ATPase complex assembly |  |
| CI | rs61560 | 1B | [TraesCS1B02G308700](http://plants.ensembl.org/Triticum_aestivum/Gene/Summary?db=core;g=TraesCS1B02G308700;tl=KFwyAitabYJE9bzD-21600162-2334044931) | [531206120-531206167](http://plants.ensembl.org/Triticum_aestivum/Location/View?r=1B:531206117-531206169;tl=KFwyAitabYJE9bzD-21600162-2334044931) |  | protein binding, zinc ion binding |
| FDS | rs61560 | 1B | [TraesCS1B02G308700](http://plants.ensembl.org/Triticum_aestivum/Gene/Summary?db=core;g=TraesCS1B02G308700;tl=vH5HPt9IHWDNYPYE-21600914-2334181889) | [531206120-531206167](http://plants.ensembl.org/Triticum_aestivum/Location/View?r=1B:531206117-531206169;tl=vH5HPt9IHWDNYPYE-21600914-2334181889) |  |  |
| LR_98_22 | rs49543 | 1B | [TraesCS1B02G326700](http://plants.ensembl.org/Triticum_aestivum/Gene/Summary?db=core;g=TraesCS1B02G326700;tl=ZhHx4efq1ggOWiPn-21600974-2334192438) | [553020392-553020415](http://plants.ensembl.org/Triticum_aestivum/Location/View?r=1B:553020390-553020416;tl=ZhHx4efq1ggOWiPn-21600974-2334192438) |  | RNA polymerase III general transcription initiation factor activity, DNA binding |
| LR_99_2 | rs39239 | 2A | [TraesCS2A02G002100](http://plants.ensembl.org/Triticum_aestivum/Gene/Summary?db=core;g=TraesCS2A02G002100;tl=oOzs4QQ4vqJc1dl5-21594918-2333353100) | [1165171-1165202](http://plants.ensembl.org/Triticum_aestivum/Location/View?r=2A:1165169-1165203;tl=oOzs4QQ4vqJc1dl5-21594918-2333353100) |  | protein binding |
| LR_99_2 | rs45212 | 2A | [TraesCS2A02G075200](http://plants.ensembl.org/Triticum_aestivum/Gene/Summary?db=core;g=TraesCS2A02G075200;tl=oOzs4QQ4vqJc1dl5-21594919-2333353121) | [33335192-33335229](http://plants.ensembl.org/Triticum_aestivum/Location/View?r=2A:33335190-33335230;tl=oOzs4QQ4vqJc1dl5-21594919-2333353121) |  | catalytic activity, transketolase activity |
| AUPPC | rs15134 | 2A | [TraesCS2A02G226800](http://plants.ensembl.org/Triticum_aestivum/Gene/Summary?db=core;g=TraesCS2A02G226800;tl=oOzs4QQ4vqJc1dl5-21594924-2333354434) | [236832962-236833024](http://plants.ensembl.org/Triticum_aestivum/Location/View?r=2A:236832958-236833027;tl=oOzs4QQ4vqJc1dl5-21594924-2333354434) |  | peptidyl-prolyl cis-trans isomerase activity |
| CI | rs15134 | 2A | [TraesCS2A02G226800](https://plants.ensembl.org/Triticum_aestivum/Gene/Summary?db=core;g=TraesCS2A02G226800;tl=oUt0aOmnmbJ3DlhC-21595053-2333379001) | [236832962-236833024](https://plants.ensembl.org/Triticum_aestivum/Location/View?r=2A:236832958-236833027;tl=oUt0aOmnmbJ3DlhC-21595053-2333379001) |  | peptidyl-prolyl cis-trans isomerase activity |
| FDS | rs15134 | 2A | [TraesCS2A02G226800](http://plants.ensembl.org/Triticum_aestivum/Gene/Summary?db=core;g=TraesCS2A02G226800;tl=KFwyAitabYJE9bzD-21600169-2334044977) | [236832962-236833024](http://plants.ensembl.org/Triticum_aestivum/Location/View?r=2A:236832958-236833027;tl=KFwyAitabYJE9bzD-21600169-2334044977) |  | peptidyl-prolyl cis-trans isomerase activity |
| LR_98_22 | rs15134 | 2A | [TraesCS2A02G226800](https://plants.ensembl.org/Triticum_aestivum/Gene/Summary?db=core;g=TraesCS2A02G226800;tl=7Ww4HO1DFSSlH0HF-21600591-2334118363) | [236832962-236833024](https://plants.ensembl.org/Triticum_aestivum/Location/View?r=2A:236832958-236833027;tl=7Ww4HO1DFSSlH0HF-21600591-2334118363) |  | peptidyl-prolyl cis-trans isomerase activity |
| FDS | rs12012 | 2A | [TraesCS2A02G249700](http://plants.ensembl.org/Triticum_aestivum/Gene/Summary?db=core;g=TraesCS2A02G249700;tl=KFwyAitabYJE9bzD-21600168-2334046367) | [376085092-376085152](http://plants.ensembl.org/Triticum_aestivum/Location/View?r=2A:376085089-376085155;tl=KFwyAitabYJE9bzD-21600168-2334046367) |  | hydrolase activity, acting on ester bonds |
| AUPPC | rs13733 | 2A | [TraesCS2A02G249700](http://plants.ensembl.org/Triticum_aestivum/Gene/Summary?db=core;g=TraesCS2A02G249700;tl=oOzs4QQ4vqJc1dl5-21594923-2333353649) | [376085092-376085152](http://plants.ensembl.org/Triticum_aestivum/Location/View?r=2A:376085089-376085155;tl=oOzs4QQ4vqJc1dl5-21594923-2333353649) |  | hydrolase activity, acting on ester bonds |
| CI | rs13733 | 2A | [TraesCS2A02G249700](http://plants.ensembl.org/Triticum_aestivum/Gene/Summary?db=core;g=TraesCS2A02G249700;tl=5I3PTjNHweDYsH7r-21594996-2333378491) | [376085092-376085152](http://plants.ensembl.org/Triticum_aestivum/Location/View?r=2A:376085089-376085155;tl=5I3PTjNHweDYsH7r-21594996-2333378491) |  | hydrolase activity, acting on ester bonds |
| LR_98_12 | rs42326 | 2A | TraesCS2A02G590400   TraesCS2A02G590300 | [778602117-778602138](http://plants.ensembl.org/Triticum_aestivum/Location/View?r=2A:778602115-778602139;tl=HLmXJZDxIN3j6Db0-21600775-2334161420) | transmembrane transport | transmembrane transporter activity |
| LR_97_12 | rs53036 | 2B | [TraesCS2B02G040200](http://plants.ensembl.org/Triticum_aestivum/Gene/Summary?db=core;g=TraesCS2B02G040200;tl=ZhHx4efq1ggOWiPn-21600988-2334194852) | [18261608-18261643](http://plants.ensembl.org/Triticum_aestivum/Location/View?r=2B:18261606-18261644;tl=ZhHx4efq1ggOWiPn-21600988-2334194852) |  |  |
| LR_97_12 | rs2483 | 2B | TraesCS2B02G043100 | [19,968,050-19,971,578](http://plants.ensembl.org/Triticum_aestivum/Location/View?db=core;g=TraesCS2B02G043100;r=2B:19968050-19971578;t=TraesCS2B02G043100.1;tl=w7GBhUA5I1qEXKJ8-21592924-2333102220) | exocytosis, protein transport |  |
| LR_97_12 | rs1919 | 2B | TraesCS2B02G396000 | 562,217,205-562,219,581 |  | oxidoreductase activity, metal ion binding |
| LR_99_2 | rs750 | 2B | [TraesCS2B02G477000](http://plants.ensembl.org/Triticum_aestivum/Gene/Summary?db=core;g=TraesCS2B02G477000;tl=Vmar1yCccMzkV0ck-21600188-2334047981) | [673961541-673961604](http://plants.ensembl.org/Triticum_aestivum/Location/View?r=2B:673961537-673961607;tl=Vmar1yCccMzkV0ck-21600188-2334047981) |  |  |
| AUPPC | rs5799 | 2B | [TraesCS2B02G537000](http://plants.ensembl.org/Triticum_aestivum/Gene/Summary?db=core;g=TraesCS2B02G537000;tl=5I3PTjNHweDYsH7r-21594982-2333372312) | [732348375-732348438](http://plants.ensembl.org/Triticum_aestivum/Location/View?r=2B:732348371-732348441;tl=5I3PTjNHweDYsH7r-21594982-2333372312) |  |  |
| AUPPC | rs5800 | 2B | [TraesCS2B02G537000](http://plants.ensembl.org/Triticum_aestivum/Gene/Summary?db=core;g=TraesCS2B02G537000;tl=5I3PTjNHweDYsH7r-21594982-2333372312) | [732348375-732348438](http://plants.ensembl.org/Triticum_aestivum/Location/View?r=2B:732348371-732348441;tl=5I3PTjNHweDYsH7r-21594982-2333372312) |  |  |
| CI | rs5800 | 2B | [TraesCS2B02G537000](http://plants.ensembl.org/Triticum_aestivum/Gene/Summary?db=core;g=TraesCS2B02G537000;tl=KFwyAitabYJE9bzD-21600156-2334044786) | [732348375-732348438](http://plants.ensembl.org/Triticum_aestivum/Location/View?r=2B:732348371-732348441;tl=KFwyAitabYJE9bzD-21600156-2334044786) |  |  |
| AUDPC | rs9382 | 2B | TraesCS2B02G537000 | :732350818-732350881 |  |  |
| FDS | rs5800 | 2B | [TraesCS2B02G537000](http://plants.ensembl.org/Triticum_aestivum/Gene/Summary?db=core;g=TraesCS2B02G537000;tl=Vmar1yCccMzkV0ck-21600206-2334054860) | [732348375-732348438](http://plants.ensembl.org/Triticum_aestivum/Location/View?r=2B:732348371-732348441;tl=Vmar1yCccMzkV0ck-21600206-2334054860) |  |  |
| AUPPC | rs34865 | 2B | [TraesCS2B02G623300](http://plants.ensembl.org/Triticum_aestivum/Gene/Summary?db=core;g=TraesCS2B02G623300;tl=5I3PTjNHweDYsH7r-21594973-2333371991) | [797110730-797110793](http://plants.ensembl.org/Triticum_aestivum/Location/View?r=2B:797110726-797110796;tl=5I3PTjNHweDYsH7r-21594973-2333371991) |  |  |
| FDS | rs34865 | 2B | [TraesCS2B02G623300](http://plants.ensembl.org/Triticum_aestivum/Gene/Summary?db=core;g=TraesCS2B02G623300;tl=iHjZi4TBKRIemlUU-21600738-2334147556) | [797110730-797110793](http://plants.ensembl.org/Triticum_aestivum/Location/View?r=2B:797110726-797110796;tl=iHjZi4TBKRIemlUU-21600738-2334147556) |  |  |
| LR_98_12 | rs62734 | 2D | [TraesCS2D02G046900](http://plants.ensembl.org/Triticum_aestivum/Gene/Summary?db=core;g=TraesCS2D02G046900;tl=vH5HPt9IHWDNYPYE-21600916-2334189097) | [17184268-17184331](http://plants.ensembl.org/Triticum_aestivum/Location/View?r=2D:17184264-17184334;tl=vH5HPt9IHWDNYPYE-21600916-2334189097) |  |  |
| FDS | rs2385 | 2D | [TraesCS2D02G465400](http://plants.ensembl.org/Triticum_aestivum/Gene/Summary?db=core;g=TraesCS2D02G465400;tl=KFwyAitabYJE9bzD-21600179-2334045566) | [571212003-571212063](http://plants.ensembl.org/Triticum_aestivum/Location/View?r=2D:571212000-571212066;tl=KFwyAitabYJE9bzD-21600179-2334045566) |  | ADP binding |
| LR_99_2 | rs59576 | 2D | [TraesCS2D02G479000](http://plants.ensembl.org/Triticum_aestivum/Gene/Summary?db=core;g=TraesCS2D02G479000;tl=vH5HPt9IHWDNYPYE-21600908-2334181845) | [580005996-580006059](http://plants.ensembl.org/Triticum_aestivum/Location/View?r=2D:580005992-580006062;tl=vH5HPt9IHWDNYPYE-21600908-2334181845) |  |  |
| LR_97_12 | rs7934 | 3A | [TraesCS3A02G013400](https://plants.ensembl.org/Triticum_aestivum/Gene/Summary?db=core;g=TraesCS3A02G013400;tl=y4vwUXp8q7a5j3ZF-21600230-2334055222) | [9783188-9783251](https://plants.ensembl.org/Triticum_aestivum/Location/View?r=3A:9783184-9783254;tl=y4vwUXp8q7a5j3ZF-21600230-2334055222) | protein phosphorylation | protein kinase activity, ATP binding |
| LR_99_2 | rs26180 | 3A | [TraesCS3A02G280100](http://plants.ensembl.org/Triticum_aestivum/Gene/Summary?db=core;g=TraesCS3A02G280100;tl=q3Kh0T9JDxos5Qcr-21600627-2334135880) | [509407655-509407693](http://plants.ensembl.org/Triticum_aestivum/Location/View?r=3A:509407653-509407694;tl=q3Kh0T9JDxos5Qcr-21600627-2334135880) |  |  |
| AUDPC | rs9410 | 3B | TraesCS3B02G070400 | [42369235-42369294](http://plants.ensembl.org/Triticum_aestivum/Location/View?r=3B:42369232-42369296;tl=WXnFacBUYZeZAPzk-21592883-2333100700) | regulation of transcription, DNA-templated | DNA-binding transcription factor activity, sequence-specific DNA binding, protein dimerization activity |
| LR_97_12 | rs46842 | 3B | [TraesCS3B02G102600](http://plants.ensembl.org/Triticum_aestivum/Gene/Summary?db=core;g=TraesCS3B02G102600;tl=w7GBhUA5I1qEXKJ8-21592934-2333104515) | [69358420-69358480](http://plants.ensembl.org/Triticum_aestivum/Location/View?r=3B:69358417-69358483;tl=w7GBhUA5I1qEXKJ8-21592934-2333104515) |  | oxidoreductase activity, oxidoreductase activity, acting on the CH-CH group of donors, NAD or NADP as acceptor, metal ion binding |
| LR_97_12 | rs2021 | 3B | TraesCS3B02G450500 | 690877996-690878058 |  |  |
| LR_98_12 | rs1653 | 3B | [TraesCS3B02G582400](http://plants.ensembl.org/Triticum_aestivum/Gene/Summary?db=core;g=TraesCS3B02G582400;tl=Vmar1yCccMzkV0ck-21600191-2334050984) | [810534237-810534259](http://plants.ensembl.org/Triticum_aestivum/Location/View?r=3B:810534235-810534260;tl=Vmar1yCccMzkV0ck-21600191-2334050984) | transcription open complex formation at RNA polymerase II promoter, regulation of transcription, DNA-templated, transcription initiation from RNA polymerase II promoter, | sequence-specific DNA binding |
| LR_98_12 | rs10220 | 3D | [TraesCS3D02G088400](https://plants.ensembl.org/Triticum_aestivum/Gene/Summary?db=core;g=TraesCS3D02G088400;tl=y4vwUXp8q7a5j3ZF-21600238-2334055274) | [45029418-45029481](https://plants.ensembl.org/Triticum_aestivum/Location/View?r=3D:45029414-45029484;tl=y4vwUXp8q7a5j3ZF-21600238-2334055274) | response to wounding, negative regulation of peptidase activity, negative regulation of endopeptidase activity | serine-type endopeptidase inhibitor activity, peptidase inhibitor activity |
| LR_98_12 | rs62871 | 3D | [TraesCS3D02G089600](http://plants.ensembl.org/Triticum_aestivum/Gene/Summary?db=core;g=TraesCS3D02G089600;tl=vH5HPt9IHWDNYPYE-21600917-2334181900) | [45406609-45406672](http://plants.ensembl.org/Triticum_aestivum/Location/View?r=3D:45406605-45406675;tl=vH5HPt9IHWDNYPYE-21600917-2334181900) |  |  |
| LR_98_12 | rs14417 | 3D | [TraesCS3D02G090300](https://plants.ensembl.org/Triticum_aestivum/Gene/Summary?db=core;g=TraesCS3D02G090300;tl=y4vwUXp8q7a5j3ZF-21600255-2334098530) | [45923249-45923290](https://plants.ensembl.org/Triticum_aestivum/Location/View?r=3D:45923246-45923292;tl=y4vwUXp8q7a5j3ZF-21600255-2334098530) | regulation of transcription, DNA-templated | DNA binding, DNA-binding transcription factor activity, protein dimerization activity |
| FDS | rs784 | 4A | [TraesCS4A02G005200](http://plants.ensembl.org/Triticum_aestivum/Gene/Summary?db=core;g=TraesCS4A02G005200;tl=WXnFacBUYZeZAPzk-21592897-2333101284) | [3220558-3220621](http://plants.ensembl.org/Triticum_aestivum/Location/View?r=4A:3220554-3220624;tl=WXnFacBUYZeZAPzk-21592897-2333101284) |  |  |
| LR_98_22 | rs62568 | 4A | [TraesCS4A02G163700](http://plants.ensembl.org/Triticum_aestivum/Gene/Summary?db=core;g=TraesCS4A02G163700;tl=w7GBhUA5I1qEXKJ8-21592950-2333120452) | [371791702-371791765](http://plants.ensembl.org/Triticum_aestivum/Location/View?r=4A:371791698-371791768;tl=w7GBhUA5I1qEXKJ8-21592950-2333120452) | inositol biosynthetic process, response to cold, L-ascorbic acid biosynthetic process, phosphatidylinositol phosphate biosynthetic process, inositol phosphate dephosphorylation, response to karrikin | inositol monophosphate 1-phosphatase activity, L-galactose-1-phosphate phosphatase activity, metal ion binding, inositol monophosphate 3-phosphatase activity, inositol monophosphate 4-phosphatase activity |
| LR_98_12 | rs15492 | 4A | [TraesCS4A02G256700](https://plants.ensembl.org/Triticum_aestivum/Gene/Summary?db=core;g=TraesCS4A02G256700;tl=7Ww4HO1DFSSlH0HF-21600592-2334118863) | [569477689-569477706](https://plants.ensembl.org/Triticum_aestivum/Location/View?r=4A:569477688-569477706;tl=7Ww4HO1DFSSlH0HF-21600592-2334118863) | regulation of transcription, DNA-templated, regulation of transcription by RNA polymerase II | DNA-binding transcription factor activity, RNA polymerase II-specific, DNA binding, DNA-binding transcription factor activity, sequence-specific DNA binding |
| LR_98_22 | rs372 | 4A | [TraesCS4A02G315100](http://plants.ensembl.org/Triticum_aestivum/Gene/Summary?db=core;g=TraesCS4A02G315100;tl=Vmar1yCccMzkV0ck-21600185-2334050054) | [605575954-605576017](http://plants.ensembl.org/Triticum_aestivum/Location/View?r=4A:605575950-605576020;tl=Vmar1yCccMzkV0ck-21600185-2334050054) | protein phosphorylation |  |
| LR_97_12 | rs53835 | 4A | [TraesCS4A02G414600](http://plants.ensembl.org/Triticum_aestivum/Gene/Summary?db=core;g=TraesCS4A02G414600;tl=ZhHx4efq1ggOWiPn-21600991-2334193943) | [684909652-684909715](http://plants.ensembl.org/Triticum_aestivum/Location/View?r=4A:684909648-684909718;tl=ZhHx4efq1ggOWiPn-21600991-2334193943) | protein phosphorylation, male meiosis cytokinesis, lateral root formation, phosphorylation | nucleotide binding, protein kinase activity, protein serine/threonine kinase activity, MAP kinase kinase activity, ATP binding, kinase activity, transferase activity |
| LR_97_12 | rs2007 | 4A | [TraesCS4A02G441100](http://plants.ensembl.org/Triticum_aestivum/Gene/Summary?db=core;g=TraesCS4A02G441100;tl=Vmar1yCccMzkV0ck-21600193-2334049038) | [709857646-709857703](http://plants.ensembl.org/Triticum_aestivum/Location/View?r=4A:709857643-709857705;tl=Vmar1yCccMzkV0ck-21600193-2334049038) |  |  |
| AUPPC | rs9745 | 4B | [TraesCS4B02G359100](http://plants.ensembl.org/Triticum_aestivum/Gene/Summary?db=core;g=TraesCS4B02G359100;tl=5I3PTjNHweDYsH7r-21594994-2333377868) | [649542814-649542877](http://plants.ensembl.org/Triticum_aestivum/Location/View?r=4B:649542810-649542880;tl=5I3PTjNHweDYsH7r-21594994-2333377868) |  | protein binding |
| LR_99_2 | rs44096 | 4B | [TraesCS4B02G360500](http://plants.ensembl.org/Triticum_aestivum/Gene/Summary?db=core;g=TraesCS4B02G360500;tl=HLmXJZDxIN3j6Db0-21600779-2334161470) | [650632796-650632843](http://plants.ensembl.org/Triticum_aestivum/Location/View?r=4B:650632793-650632845;tl=HLmXJZDxIN3j6Db0-21600779-2334161470) | GDP-L-fucose salvage | ATP binding, transferase activity, transferring phosphorus-containing groups, fucose-1-phosphate guanylyltransferase activity, fucokinase activity |
| LR_98_12 | rs58692 | 4D | [TraesCS4D02G350500](http://plants.ensembl.org/Triticum_aestivum/Gene/Summary?db=core;g=TraesCS4D02G350500;tl=w7GBhUA5I1qEXKJ8-21592942-2333104540) | [502710747-502710810](http://plants.ensembl.org/Triticum_aestivum/Location/View?r=4D:502710743-502710813;tl=w7GBhUA5I1qEXKJ8-21592942-2333104540) | carbohydrate metabolic process, metabolic process | hydrolase activity, hydrolyzing O-glycosyl compounds, beta-fructofuranosidase activity, hydrolase activity, hydrolase activity, acting on glycosyl bonds |
| LR_99_2 | rs58692 | 4D | [TraesCS4D02G350500](http://plants.ensembl.org/Triticum_aestivum/Gene/Summary?db=core;g=TraesCS4D02G350500;tl=oOzs4QQ4vqJc1dl5-21594922-2333353150) | [502710747-502710810](http://plants.ensembl.org/Triticum_aestivum/Location/View?r=4D:502710743-502710813;tl=oOzs4QQ4vqJc1dl5-21594922-2333353150) | carbohydrate metabolic process, metabolic process | hydrolase activity, hydrolyzing O-glycosyl compounds, beta-fructofuranosidase activity, hydrolase activity, hydrolase activity, acting on glycosyl bonds |
| LR_98_12 | rs10978 | 5A | [TraesCS5A02G392100](https://plants.ensembl.org/Triticum_aestivum/Gene/Summary?db=core;g=TraesCS5A02G392100;tl=y4vwUXp8q7a5j3ZF-21600240-2334088607) | [588416892-588416939](https://plants.ensembl.org/Triticum_aestivum/Location/View?r=5A:588416889-588416941;tl=y4vwUXp8q7a5j3ZF-21600240-2334088607) |  |  |
| LR_97_12 | rs43827 | 5A | [TraesCS5A02G439600](http://plants.ensembl.org/Triticum_aestivum/Gene/Summary?db=core;g=TraesCS5A02G439600;tl=HLmXJZDxIN3j6Db0-21600776-2334161439) | [621009948-621009973](http://plants.ensembl.org/Triticum_aestivum/Location/View?r=5A:621009946-621009974;tl=HLmXJZDxIN3j6Db0-21600776-2334161439) |  |  |
| LR_98_22 | rs51983 | 5A | [TraesCS5A02G515300](http://plants.ensembl.org/Triticum_aestivum/Gene/Summary?db=core;g=TraesCS5A02G515300;tl=ZhHx4efq1ggOWiPn-21600984-2334192596) | [678624200-678624263](http://plants.ensembl.org/Triticum_aestivum/Location/View?r=5A:678624196-678624266;tl=ZhHx4efq1ggOWiPn-21600984-2334192596) |  |  |
| CI | rs31181 | 5B | [TraesCS5B02G024300](http://plants.ensembl.org/Triticum_aestivum/Gene/Summary?db=core;g=TraesCS5B02G024300;tl=WXnFacBUYZeZAPzk-21592885-2333100714) | [22513839-22513902](http://plants.ensembl.org/Triticum_aestivum/Location/View?r=5B:22513835-22513905;tl=WXnFacBUYZeZAPzk-21592885-2333100714) |  |  |
| LR_98_12 | rs36886 | 5B | [TraesCS5B02G079300](http://plants.ensembl.org/Triticum_aestivum/Gene/Summary?db=core;g=TraesCS5B02G079300;tl=iHjZi4TBKRIemlUU-21600753-2334147754) | [96757160-96757178](http://plants.ensembl.org/Triticum_aestivum/Location/View?r=5B:96757159-96757178;tl=iHjZi4TBKRIemlUU-21600753-2334147754) |  | nucleic acid binding, RNA helicase activity, ATP binding, |
| FDS | rs50947 | 5B | TraesCS5B02G166400 | 308,556,184-308,567,925 |  | catalytic activity |
| LR_98_12 | rs36084 | 5B | [TraesCS5B02G273100](http://plants.ensembl.org/Triticum_aestivum/Gene/Summary?db=core;g=TraesCS5B02G273100;tl=iHjZi4TBKRIemlUU-21600746-2334157292) | [458663129-458663192](http://plants.ensembl.org/Triticum_aestivum/Location/View?r=5B:458663125-458663195;tl=iHjZi4TBKRIemlUU-21600746-2334157292) |  |  |
| LR_99_2 | rs47218 | 5B | [TraesCS5B02G385300](http://plants.ensembl.org/Triticum_aestivum/Gene/Summary?db=core;g=TraesCS5B02G385300;tl=HLmXJZDxIN3j6Db0-21600792-2334169901) | [564988668-564988709](http://plants.ensembl.org/Triticum_aestivum/Location/View?r=5B:564988665-564988711;tl=HLmXJZDxIN3j6Db0-21600792-2334169901) |  |  |
| LR_98_12 | rs57916 | 5B | [TraesCS5B02G415400](http://plants.ensembl.org/Triticum_aestivum/Gene/Summary?db=core;g=TraesCS5B02G415400;tl=ZhHx4efq1ggOWiPn-21601001-2334194082) | [589677101-589677164](http://plants.ensembl.org/Triticum_aestivum/Location/View?r=5B:589677097-589677167;tl=ZhHx4efq1ggOWiPn-21601001-2334194082) | regulation of transcription, DNA-templated | DNA binding |
| AUPPC | rs6872 | 5B | [TraesCS5B02G501400](http://plants.ensembl.org/Triticum_aestivum/Gene/Summary?db=core;g=TraesCS5B02G501400;tl=5I3PTjNHweDYsH7r-21594991-2333377797) | [668582446-668582478](http://plants.ensembl.org/Triticum_aestivum/Location/View?r=5B:668582444-668582479;tl=5I3PTjNHweDYsH7r-21594991-2333377797) |  | aspartic-type endopeptidase activity |
| CI | rs6872 | 5B | [TraesCS5B02G501400](http://plants.ensembl.org/Triticum_aestivum/Gene/Summary?db=core;g=TraesCS5B02G501400;tl=KFwyAitabYJE9bzD-21600165-2334045671) | [668582446-668582478](http://plants.ensembl.org/Triticum_aestivum/Location/View?r=5B:668582444-668582479;tl=KFwyAitabYJE9bzD-21600165-2334045671) |  | aspartic-type endopeptidase activity |
| FDS | rs6872 | 5B | [TraesCS5B02G501400](https://plants.ensembl.org/Triticum_aestivum/Gene/Summary?db=core;g=TraesCS5B02G501400;tl=y4vwUXp8q7a5j3ZF-21600227-2334055158) | [668582446-668582478](https://plants.ensembl.org/Triticum_aestivum/Location/View?r=5B:668582444-668582479;tl=y4vwUXp8q7a5j3ZF-21600227-2334055158) |  | aspartic-type endopeptidase activity |
| LR_97_12 | rs1798 | 5D | [TraesCS5D02G361200](http://plants.ensembl.org/Triticum_aestivum/Gene/Summary?db=core;g=TraesCS5D02G361200;tl=Vmar1yCccMzkV0ck-21600192-2334052261) | [440735849-440735912](http://plants.ensembl.org/Triticum_aestivum/Location/View?r=5D:440735845-440735915;tl=Vmar1yCccMzkV0ck-21600192-2334052261) |  |  |
| LR_97_12 | rs27896 | 5D | [TraesCS5D02G502400](http://plants.ensembl.org/Triticum_aestivum/Gene/Summary?db=core;g=TraesCS5D02G502400;tl=w7GBhUA5I1qEXKJ8-21592925-2333118108) | [529836240-529836276](http://plants.ensembl.org/Triticum_aestivum/Location/View?r=5D:529836238-529836277;tl=w7GBhUA5I1qEXKJ8-21592925-2333118108) |  | protein binding |
| CI | rs34317 | 6A | [TraesCS6A02G191000](https://plants.ensembl.org/Triticum_aestivum/Gene/Summary?db=core;g=TraesCS6A02G191000;tl=oUt0aOmnmbJ3DlhC-21595076-2333386091) | [256483195-256483212](https://plants.ensembl.org/Triticum_aestivum/Location/View?r=6A:256483194-256483212;tl=oUt0aOmnmbJ3DlhC-21595076-2333386091) | protein phosphorylation | protein kinase activity, phosphoprotein phosphatase activity, ATP binding, phosphatase activity, cation binding, metal ion binding |
| LR_97_12 | rs48221 | 6A | [TraesCS6A02G342000](http://plants.ensembl.org/Triticum_aestivum/Gene/Summary?db=core;g=TraesCS6A02G342000;tl=w7GBhUA5I1qEXKJ8-21592935-2333092834) | [574526056-574526118](http://plants.ensembl.org/Triticum_aestivum/Location/View?r=6A:574526052-574526121;tl=w7GBhUA5I1qEXKJ8-21592935-2333092834) | protein phosphorylation | protein kinase activity, calcium ion binding, ATP binding, polysaccharide binding |
| LR_99_2 | rs53897 | 6A | [TraesCS6A02G378500](http://plants.ensembl.org/Triticum_aestivum/Gene/Summary?db=core;g=TraesCS6A02G378500;tl=ZhHx4efq1ggOWiPn-21600992-2334192932) | [599261582-599261645](http://plants.ensembl.org/Triticum_aestivum/Location/View?r=6A:599261578-599261648;tl=ZhHx4efq1ggOWiPn-21600992-2334192932) | regulation of transcription, DNA-templated | DNA binding |
| CI | rs46898 | 6A | [TraesCS6A02G411800](http://plants.ensembl.org/Triticum_aestivum/Gene/Summary?db=core;g=TraesCS6A02G411800;tl=WXnFacBUYZeZAPzk-21592887-2333100737) | [613963677-613963738](http://plants.ensembl.org/Triticum_aestivum/Location/View?r=6A:613963673-613963741;tl=WXnFacBUYZeZAPzk-21592887-2333100737) | ADP binding |  |
| LR_99_2 | rs44700 | 6A | [TraesCS6A02G419200](http://plants.ensembl.org/Triticum_aestivum/Gene/Summary?db=core;g=TraesCS6A02G419200;tl=HLmXJZDxIN3j6Db0-21600782-2334170969) | [616823445-616823508](http://plants.ensembl.org/Triticum_aestivum/Location/View?r=6A:616823441-616823511;tl=HLmXJZDxIN3j6Db0-21600782-2334170969) | protein ubiquitination | ubiquitin-protein transferase activity, protein binding, transferase activity, ligase activity |
| AUPPC | rs37116 | 6B | TraesCS6B02G125200 | [120318178-120318241](http://plants.ensembl.org/Triticum_aestivum/Location/View?r=6B:120318174-120318244;tl=5I3PTjNHweDYsH7r-21594974-2333373373) | SCF-dependent proteasomal ubiquitin-dependent protein catabolic process | protein binding |
| CI | rs37116 | 6B | [TraesCS6B02G125200](https://plants.ensembl.org/Triticum_aestivum/Gene/Summary?db=core;g=TraesCS6B02G125200;tl=oUt0aOmnmbJ3DlhC-21595077-2333386183) | [120318178-120318241](https://plants.ensembl.org/Triticum_aestivum/Location/View?r=6B:120318174-120318244;tl=oUt0aOmnmbJ3DlhC-21595077-2333386183) | SCF-dependent proteasomal ubiquitin-dependent protein catabolic process | protein binding |
| AUPPC | rs20851 | 6B | [TraesCS6B02G170200](http://plants.ensembl.org/Triticum_aestivum/Gene/Summary?db=core;g=TraesCS6B02G170200;tl=oOzs4QQ4vqJc1dl5-21594929-2333353177) | [182585584-182585647](http://plants.ensembl.org/Triticum_aestivum/Location/View?r=6B:182585580-182585650;tl=oOzs4QQ4vqJc1dl5-21594929-2333353177) | lipid metabolic process, lipid catabolic process | hydrolase activity |
| CI | rs20851 | 6B | [TraesCS6B02G170200](https://plants.ensembl.org/Triticum_aestivum/Gene/Summary?db=core;g=TraesCS6B02G170200;tl=oUt0aOmnmbJ3DlhC-21595058-2333380896) | [182585584-182585647](https://plants.ensembl.org/Triticum_aestivum/Location/View?r=6B:182585580-182585650;tl=oUt0aOmnmbJ3DlhC-21595058-2333380896) | lipid metabolic process, lipid catabolic process | hydrolase activity |
| FDS | rs20851 | 6B | [TraesCS6B02G170200](http://plants.ensembl.org/Triticum_aestivum/Gene/Summary?db=core;g=TraesCS6B02G170200;tl=KFwyAitabYJE9bzD-21600175-2334045550) | [182585584-182585647](http://plants.ensembl.org/Triticum_aestivum/Location/View?r=6B:182585580-182585650;tl=KFwyAitabYJE9bzD-21600175-2334045550) | lipid metabolic process, lipid catabolic process | hydrolase activity |
| AUDPC | rs61673 | 6B | TraesCS6B02G225400 | 346,785,014-346,790,226 | regulation of nuclear division, negative regulation of ubiquitin protein ligase activity |  |
| LR_98_12 | rs36483 | 6B | [TraesCS6B02G453300](http://plants.ensembl.org/Triticum_aestivum/Gene/Summary?db=core;g=TraesCS6B02G453300;tl=iHjZi4TBKRIemlUU-21600750-2334153373) | [711709425-711709469](http://plants.ensembl.org/Triticum_aestivum/Location/View?r=6B:711709422-711709471;tl=iHjZi4TBKRIemlUU-21600750-2334153373) |  | protein binding |
| LR_99_2 | rs41848 | 6D | [TraesCS6D02G106000](http://plants.ensembl.org/Triticum_aestivum/Gene/Summary?db=core;g=TraesCS6D02G106000;tl=HLmXJZDxIN3j6Db0-21600771-2334161279) | [69219148-69219211](http://plants.ensembl.org/Triticum_aestivum/Location/View?r=6D:69219144-69219214;tl=HLmXJZDxIN3j6Db0-21600771-2334161279) |  |  |
| LR_98_12 | rs51542 | 6D | [TraesCS6D02G106000](http://plants.ensembl.org/Triticum_aestivum/Gene/Summary?db=core;g=TraesCS6D02G106000;tl=ZhHx4efq1ggOWiPn-21600983-2334193009) | [69219088-69219151](http://plants.ensembl.org/Triticum_aestivum/Location/View?r=6D:69219084-69219154;tl=ZhHx4efq1ggOWiPn-21600983-2334193009) |  |  |
| LR_98_12 | rs184 | 6D | [TraesCS6D02G111700](http://plants.ensembl.org/Triticum_aestivum/Gene/Summary?db=core;g=TraesCS6D02G111700;tl=Vmar1yCccMzkV0ck-21600184-2334047954) | [78272691-78272712](http://plants.ensembl.org/Triticum_aestivum/Location/View?r=6D:78272689-78272713;tl=Vmar1yCccMzkV0ck-21600184-2334047954) |  | nucleic acid binding |
| LR_99_2 | rs15009 | 7A | [TraesCS7A02G017500](http://plants.ensembl.org/Triticum_aestivum/Gene/Summary?db=core;g=TraesCS7A02G017500;tl=oOzs4QQ4vqJc1dl5-21594914-2333353013) | [7592711-7592756](http://plants.ensembl.org/Triticum_aestivum/Location/View?r=7A:7592708-7592758;tl=oOzs4QQ4vqJc1dl5-21594914-2333353013) |  |  |
| LR_98_22 | rs8470 | 7A | [TraesCS7A02G143600](https://plants.ensembl.org/Triticum_aestivum/Gene/Summary?db=core;g=TraesCS7A02G143600;tl=y4vwUXp8q7a5j3ZF-21600231-2334055436) | [94493003-94493050](https://plants.ensembl.org/Triticum_aestivum/Location/View?r=7A:94493000-94493052;tl=y4vwUXp8q7a5j3ZF-21600231-2334055436) |  | protein binding |
| LR_97_12 | rs16781 | 7A | [TraesCS7A02G529700](https://plants.ensembl.org/Triticum_aestivum/Gene/Summary?db=core;g=TraesCS7A02G529700;tl=7Ww4HO1DFSSlH0HF-21600596-2334118949) | [709266005-709266068](https://plants.ensembl.org/Triticum_aestivum/Location/View?r=7A:709266001-709266071;tl=7Ww4HO1DFSSlH0HF-21600596-2334118949) |  | protein binding |
| LR_99_2 | rs24338 | 7A | [TraesCS7A02G544300](https://plants.ensembl.org/Triticum_aestivum/Gene/Summary?db=core;g=TraesCS7A02G544300;tl=7Ww4HO1DFSSlH0HF-21600615-2334135734) | [720912670-720912731](https://plants.ensembl.org/Triticum_aestivum/Location/View?r=7A:720912666-720912734;tl=7Ww4HO1DFSSlH0HF-21600615-2334135734) |  | hydrolase activity, acting on ester bonds |
| LR_97_12 | rs42166 | 7B | [TraesCS7B02G033300](http://plants.ensembl.org/Triticum_aestivum/Gene/Summary?db=core;g=TraesCS7B02G033300;tl=HLmXJZDxIN3j6Db0-21600774-2334161396) | [33402975-33403038](http://plants.ensembl.org/Triticum_aestivum/Location/View?r=7B:33402971-33403041;tl=HLmXJZDxIN3j6Db0-21600774-2334161396) |  | protein binding |
| LR_98_22 | rs39305 | 7B | TraesCS7B02G042200, TraesCS7B02G042100 | [41525193-41525251](http://plants.ensembl.org/Triticum_aestivum/Location/View?r=7B:41525190-41525253;tl=iHjZi4TBKRIemlUU-21600764-2334155004) |  | oxidoreductase activity, metal ion binding, |
| LR_97_12 | rs45130 | 7B | [TraesCS7B02G173600](http://plants.ensembl.org/Triticum_aestivum/Gene/Summary?db=core;g=TraesCS7B02G173600;tl=HLmXJZDxIN3j6Db0-21600784-2334169880) | [244755053-244755070](http://plants.ensembl.org/Triticum_aestivum/Location/View?r=7B:244755052-244755070;tl=HLmXJZDxIN3j6Db0-21600784-2334169880) |  |  |
| AUPPC | rs5126 | 7B | [TraesCS7B02G225900](http://plants.ensembl.org/Triticum_aestivum/Gene/Summary?db=core;g=TraesCS7B02G225900;tl=5I3PTjNHweDYsH7r-21594977-2333372249) | [426526075-426526138](http://plants.ensembl.org/Triticum_aestivum/Location/View?r=7B:426526071-426526141;tl=5I3PTjNHweDYsH7r-21594977-2333372249) |  | protein binding |
| CI | rs5126 | 7B | [TraesCS7B02G225900](https://plants.ensembl.org/Triticum_aestivum/Gene/Summary?db=core;g=TraesCS7B02G225900;tl=oUt0aOmnmbJ3DlhC-21595082-2333381313) | [426526075-426526138](https://plants.ensembl.org/Triticum_aestivum/Location/View?r=7B:426526071-426526141;tl=oUt0aOmnmbJ3DlhC-21595082-2333381313) |  | protein binding |
| FDS | rs5126 | 7B | [TraesCS7B02G225900](http://plants.ensembl.org/Triticum_aestivum/Gene/Summary?db=core;g=TraesCS7B02G225900;tl=Vmar1yCccMzkV0ck-21600204-2334049116) | [426526075-426526138](http://plants.ensembl.org/Triticum_aestivum/Location/View?r=7B:426526071-426526141;tl=Vmar1yCccMzkV0ck-21600204-2334049116) |  | protein binding |
| LR_97_12 | rs45802 | 7B | [TraesCS7B02G407200](http://plants.ensembl.org/Triticum_aestivum/Gene/Summary?db=core;g=TraesCS7B02G407200;tl=HLmXJZDxIN3j6Db0-21600787-2334161592) | [676249917-676249980](http://plants.ensembl.org/Triticum_aestivum/Location/View?r=7B:676249913-676249983;tl=HLmXJZDxIN3j6Db0-21600787-2334161592) |  |  |
| LR_97_12 | rs47722 | 7B | [TraesCS7B02G407200](http://plants.ensembl.org/Triticum_aestivum/Gene/Summary?db=core;g=TraesCS7B02G407200;tl=HLmXJZDxIN3j6Db0-21600793-2334161609) | [676249917-676249980](http://plants.ensembl.org/Triticum_aestivum/Location/View?r=7B:676249913-676249983;tl=HLmXJZDxIN3j6Db0-21600793-2334161609) |  | magnesium ion binding, terpene synthase activity, lyase activity |
| LR_98_12 | rs33422 | 7B | [TraesCS7B02G439400](http://plants.ensembl.org/Triticum_aestivum/Gene/Summary?db=core;g=TraesCS7B02G439400;tl=q3Kh0T9JDxos5Qcr-21600646-2334136222) | [704827383-704827446](http://plants.ensembl.org/Triticum_aestivum/Location/View?r=7B:704827379-704827449;tl=q3Kh0T9JDxos5Qcr-21600646-2334136222) |  | monooxygenase activity, iron ion binding, oxidoreductase activity, acting on paired donors, with incorporation or reduction of molecular oxygen, heme binding |
| LR_98_12 | rs30371 | 7B | [TraesCS7B02G470800](http://plants.ensembl.org/Triticum_aestivum/Gene/Summary?db=core;g=TraesCS7B02G470800;tl=q3Kh0T9JDxos5Qcr-21600636-2334130862) | [727185620-727185650](http://plants.ensembl.org/Triticum_aestivum/Location/View?r=7B:727185618-727185651;tl=q3Kh0T9JDxos5Qcr-21600636-2334130862) |  |  |
| LR_98_12 | rs34968 | 7B | [TraesCS7B02G470800](http://plants.ensembl.org/Triticum_aestivum/Gene/Summary?db=core;g=TraesCS7B02G470800;tl=iHjZi4TBKRIemlUU-21600740-2334147517) | [727186324-727186383](http://plants.ensembl.org/Triticum_aestivum/Location/View?r=7B:727186321-727186385;tl=iHjZi4TBKRIemlUU-21600740-2334147517) |  |  |
| FDS | rs39629 | 3A | U6 | [34529097-34529151](http://plants.ensembl.org/Triticum_aestivum/Location/View?r=3A:34529094-34529153;tl=iHjZi4TBKRIemlUU-21600766-2334160929) |  |  |

**Table S11:** Comparison of most significant identified QTLs with previously published *Lr* genes or QTLs for both seedling and adult plant resistance to leaf rust.

| No | Trait | Marker | Chr | Physicl position (cM) | Previous marker/QTL identified | References |
| --- | --- | --- | --- | --- | --- | --- |
| 1 | AUDPC_FDS | rs20790 | 3B | 55.755 | IWA6633, IWA7889, IWA4312, Qlr.usw-3BL | 28, 61 |
| 2 | AUDPC_FDS | rs26028 | 3B | 55.755 | IWA6633, IWA7889, IWA4312, Qlr.usw-3BL | 28, 61 |
| 3 | AUDPC_FDS_CI | rs18695 | 6D | 55.761 | IWA2476 | 28 |
| 4 | AUDPC_FDS_CI | rs26220 | 1B | 66.042 | IWA3384, QLr.hebau-1BL | 28, 62 |
| 5 | AUDPC_FDS_CI | rs26318 | 2D | 81.616 | cdo1008–Xksud23a, IWA1083 | 37, 63 |
| 6 | AUDPC_FDS_CI | rs57400 | 5B | 26.242 | barc4, IWA6694, Lr52 | 64, 65 |
| 7 | AUPPC_CI | rs2383 | 2A | 74.319 | IWA5092,  IWA313, Lr38 | 28, 61, 66 |
| 8 | AUPPC_CI | rs5126 | 7B | 51.193 | QLr.hwwg-7BL (IWB9496), Xgwm577–Xbarc340.2, Xwmc273c–Xwmc311, S25M15_19–Xgwm112 | 4, 63 |
| 9 | CI | rs37116 | 6B | 43.284 | IWA3131, IWA3133, IWA5785, IWA6825, IWA6826, IWA7873, IWA8192, IWA6142, IWB11702, IWB65914, IWA3699, IWA7506, Lr9 | 9,10 |
| 10 | CI | rs6872 | 5B | 127.712 | QLr.ifa-5BL, QLr.cdl-5BL, QLr.vt-5B.1, | 67 |
| 11 | CI_FDS | rs39629 | 3A | 112.886 | QYr.hebau-3AL/QLr.hebau-3AL | 68 |
| 12 | CI_FDS | rs39631 | 3A | 112.886 | QYr.hebau-3AL/QLr.hebau-3AL | 68 |
| 13 | FDS | rs26889 | 2B | 60.32 | IWA2025, IWA2509, IWA207 | 28 |
| 14 | FDS | rs39630 | 3A | 112.886 | QYr.hebau-3AL/QLr.hebau-3AL | 68 |
| 15 | FDS_CI | rs33218 | 3B | 54.619 | IWA6633, IWA7889, IWA4312, Qlr.usw-3BL | 28, 61 |
| 16 | LR_97_12 | rs13728 | 7A | 63.946 | IWA4175 | 28 |
| 17 | LR_97_12 | rs15875 | 6B | 85.356 | IWB6474, Lr3 | 10 |
| 18 | LR_97_12 | rs15876 | 6B | 85.356 | IWB6474, Lr3 | 10 |
| 19 | LR_97_12 | rs16481 | 2D | 13.642 | Lr39, Lr22a, Lr15, Lr2a, Lr2b, Lr2c,  QLr.stars-2DS1 | 37, 69 |
| 20 | LR_97_12 | rs2007 | 4A | 124.821 | QLr.hebau-4AL | 70 |
| 21 | LR_97_12 | rs2008 | 4A | 124.821 | QLr.hebau-4AL | 70 |
| 22 | LR_97_12 | rs20781 | 4B | 57.77 | _ | _ |
| 23 | LR_97_12 | rs20782 | 4B | 57.77 | _ | _ |
| 24 | LR_97_12 | rs24374 | 6B | 58.062 | IWA3699, IWA7506 | 9 |
| 25 | LR_97_12 | rs24375 | 6B | 58.062 | IWA3699, IWA7506 | 9 |
| 26 | LR_97_12 | rs24376 | 6B | 58.062 | IWA3699, IWA7506 | 9 |
| 27 | LR_97_12 | rs32284 | 1B | 91.068 | QLr.stars-1BL1 (IWA8153), IWA6512 | 9, 37 |
| 28 | LR_97_12 | rs32285 | 1B | 91.068 | QLr.stars-1BL1 (IWA8153), IWA6512 | 9, 37 |
| 29 | LR_97_12 | rs33082 | 7B | 93.505 | Lr68, LrBi16 | 71 |
| 30 | LR_97_12 | rs33083 | 7B | 93.505 | Lr68, LrBi16 | 71 |
| 31 | LR_97_12 | rs34816 | 5B | 38.769 | IWA6694, Lr52 | 9, 65 |
| 32 | LR_97_12 | rs34817 | 5B | 38.769 | IWA6694, Lr52 | 9, 65 |
| 33 | LR_97_12 | rs39680 | 2B | 66.573 | QLr.stars-2BL1, Lr50, Lr58 | 37-39 |
| 34 | LR_97_12 | rs41570 | 6B | 62.609 | IWA3699, IWA7506 | 9 |
| 35 | LR_97_12 | rs41571 | 6B | 62.609 | IWA3699, IWA7506 | 9 |
| 36 | LR_97_12 | rs45802 | 7B | 92.919 | Lr68, LrBi16 | 71 |
| 37 | LR_97_12 | rs4602 | 5B | 35.928 | IWA6694, Lr52 | 9, 65 |
| 38 | LR_97_12 | rs47722 | 7B | 92.332 | Lr68, LrBi16 | 71 |
| 39 | LR_97_12 | rs48776 | 5B | 62.719 | IWA8375, IWA8395 | 9 |
| 40 | LR_97_12 | rs4961 | 6B | 62.609 | IWA3699, IWA7506 | 9 |
| 41 | LR_97_12 | rs51004 | 2B | 66.004 | QLr.stars-2BL1, Lr50, Lr58 | 37-39 |
| 42 | LR_97_12 | rs51367 | 6B | 46.694 | IWA3131, IWA3133, IWA5785, IWA6825, IWA6826, IWA7873, IWA8192, IWA6142, IWB11702, IWB65914, IWA3699, IWA7506, Lr9 | 9, 10 |
| 43 | LR_97_12 | rs57797 | 6B | 62.609 | IWA3699, IWA7506 | 9 |
| 44 | LR_97_12 | rs9211 | 1B | 46.711 | IWA6758 (QLr.stars-1BL2), IWA6758 (QLr.stars-1BL3) | 37 |
| 45 | LR_97_12 | rs9212 | 1B | 46.711 | IWA6758 (QLr.stars-1BL2), IWA6758 (QLr.stars-1BL3) | 37 |
| 46 | LR_97_12, LR_98_22 | rs28795 | 2D | 45.24 | QLr.stars-2DL1 , IWA1083 | 37 |
| 47 | LR_97_12, LR_98_22 | rs53036 | 2B | 72.825 | QLr.stars-2BL1, Lr50, Lr58 | 37-39 |
| 48 | LR_98_12 | rs12518 | 7B | 50.057 | IWA5000 | 9 |
| 49 | LR_98_12 | rs25737 | 3B | 45.525 | IWA6633, IWA7889, IWA4312, Qlr.usw-3BL | 28, 61 |
| 50 | LR_98_12 | rs36084 | 5B | 45.594 | IWA6694, Lr52,  QLr.stars-5BL1 | 9, 37, 65 |
| 51 | LR_98_12 | rs36555 | 6B | 47.831 | IWA3131, IWA3133, IWA5785, IWA6825, IWA6826, IWA7873, IWA8192, IWA6142, IWB11702, IWB65914, IWA3699, IWA7506, Lr9 | 9, 10 |
| 52 | LR_98_12 | rs52542 | 7B | 114.004 | Lr68, LrBi16 | 71 |
| 53 | LR_98_12 | rs58952 | 5D | 83.13 | IWB53861, Lr1, IWA1429 | 10, 28, 72 |
| 54 | LR_98_12, LR_98_22 | rs36483 | 6B | 94.461 | IWB6474, Lr3 | 10 |
| 55 | LR_98_12, LR_98_22 | rs36484 | 6B | 94.461 | IWB6474, Lr3 | 10 |
| 56 | LR_98_12, LR_98_22 | rs50047 | 3B | 22.764 | 3B_t2, Lr27 | 9, 10 |
| 57 | LR_98_12, LR_98_22, LR_97_12, LR_99_2 | rs7934 | 3A | 9.116 | Lr63, Lr66,  IWA5977 | 9, 73, 74 |
| 58 | LR_98_22 | rs1798 | 5D | 83.13 | IWB53861, Lr1, IWA1429 | 10, 28, 72 |
| 59 | LR_98_22 | rs1799 | 5D | 83.13 | IWB53861, Lr1, IWA1429 | 10, 28, 72 |
| 60 | LR_98_22 | rs37446 | 6A | 25.146 | IWA6737 | 9 |
| 61 | LR_98_22 | rs39305 | 7B | 43.237 | IWA5000 | 9 |
| 62 | LR_98_22 | rs49543 | 1B | 56.943 | _ | _ |
| 63 | LR_98_22 | rs52026 | 5A | 93.664 | _ | _ |
| 64 | LR_98_22 | rs52548 | 2B | 72.825 | QLr.stars-2BL1, Lr50, Lr58 | 37-39 |
| 65 | LR_98_22, LR98_12, LR_99_2 | rs44885 | 5D | 7.959 | _ | _ |
| 66 | LR_98_22, LR98_12, LR_99_2 | rs44886 | 5D | 7.959 | _ | _ |
| 67 | LR_99_2 | rs14658 | 4A | 76.969 | QLr.hebau-4AL | 70 |
| 68 | LR_99_2 | rs15675 | 2B | 6.832 | IWA4894,Lr13, Lr16,Lr23,Lr48,Lr73,LrZH22,LrA2K,QLr.cimmyt-2BS,QLr.hebau-2BS | 21, 70, 75-81 |
| 69 | LR_99_2 | rs15676 | 2B | 6.832 | IWA4894,Lr13, Lr16,Lr23,Lr48,Lr73,LrZH22,LrA2K,QLr.cimmyt-2BS,QLr.hebau-2BS | 21, 70, 75-81 |
| 70 | LR_99_2 | rs30506 | 1A | 80.9 | QLr.umn-1AL (IWB48030) | 10 |
| 71 | LR_99_2 | rs38255 | 3B | 113.379 | IWA6633, IWA7889, IWA4312, Qlr.usw-3BL | 28, 61 |
| 72 | LR_99_2 | rs44267 | 1B | 43.301 | IWA6758 (QLr.stars-1BL2), IWA6758 (QLr.stars-1BL3) | 37 |
| 73 | LR_99_2 | rs45341 | 3B | 113.948 | IWA6633, IWA7889, IWA4312, Qlr.usw-3BL | 28, 61 |
| 74 | LR_99_2 | rs62902 | 6B | 47.831 | IWA3131, IWA3133, IWA5785, IWA6825, IWA6826, IWA7873, IWA8192, IWA6142, IWB11702, IWB65914, IWA3699, IWA7506, Lr9 | 10, 28 |
| 75 | LR_99_2 | rs62903 | 6B | 47.831 | IWA3131, IWA3133, IWA5785, IWA6825, IWA6826, IWA7873, IWA8192, IWA6142, IWB11702, IWB65914, IWA3699, IWA7506, Lr9 | 10, 28 |
| 76 | LR_99_2 | rs6313 | 2A | 74.319 | IWA313, Lr38 | 9, 66 |
| 77 | LR_99_2 | rs6314 | 2A | 74.319 | IWA313, Lr38 | 9, 66 |
| 78 | LR_99_2 | rs750 | 2B | 67.141 | QLr.stars-2BL1, Lr50, Lr58 | 37-39 |
| 79 | LR_99_2 | rs9493 | 3B | 22.764 | 3B_t2, Lr27 | 10, 74 |
| 80 | LR_99_2, LR_97_12 | rs59576 | 2D | 73.66 | Lr54 | 73 |
