# Supplementary material for: Exploring genomic regions involved in bread wheat resistance to leaf rust at seedling/adult stages by using GWAS analysis
Source: BMC Genomics. 2023 Feb 21;24:83. doi: 10.1186/s12864-022-09096-1 (PMC9945389; doi:10.1186/s12864-022-09096-1)
Supplement: Supplementary file 3 — Additional file 3: Fig S1. Distribution of SNPs on three genomes (A) and on each chromosome (B). Fig. S2. LD decay plot of the (a) A genome, (b) B genome, (c) D genome, and (d) whole genome. Genetic distance in cM is plotted against the LD estimate (r2) for pairs of markers. The blue horizontal line indicates R2 threshold (R2=0.1), the green vertical line indicates LD decay distance, and the red line is the moving average of the 10 adjacent markers. Fig. S3. Structure plot of 320 Iranian bread wheat accessions determined by K=3 (A) and Principal component analysis (B).Fig. S4 Cluster analysis using kiniship matrix for Iranian wheat accessions. [file 12864_2022_9096_MOESM3_ESM.docx]

**Fig S1** Distribution of SNPs on three genomes (A) and on each chromosome (B)


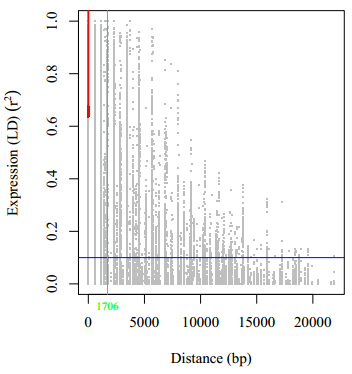

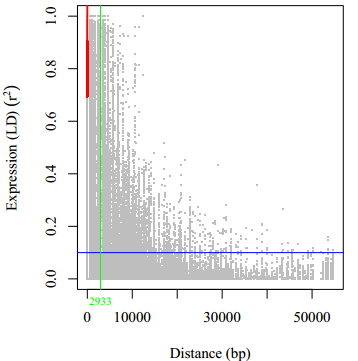

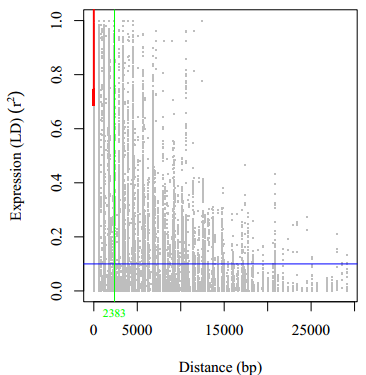

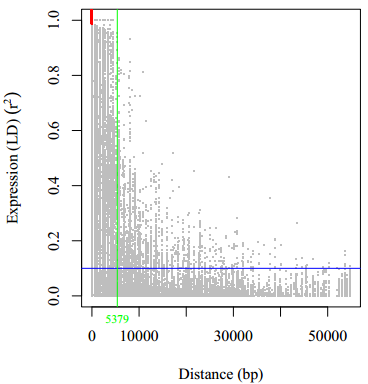


**Fig. S2** LD decay plot of the (a) A genome, (b) B genome, (c) D genome, and (d) whole genome. Genetic distance in cM is plotted against the LD estimate (r^2^) for pairs of markers. The blue horizontal line indicates R^2^ threshold (R^2^=0.1), the green vertical line indicates LD decay distance, and the red line is the moving average of the 10 adjacent markers


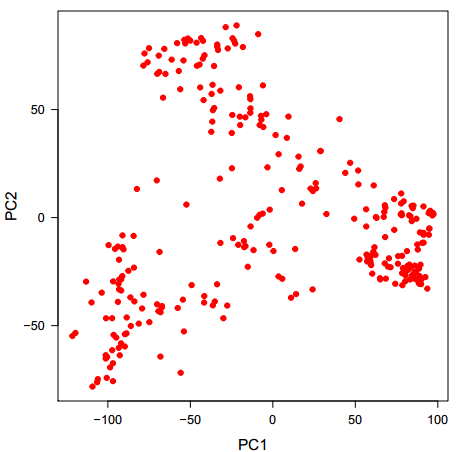

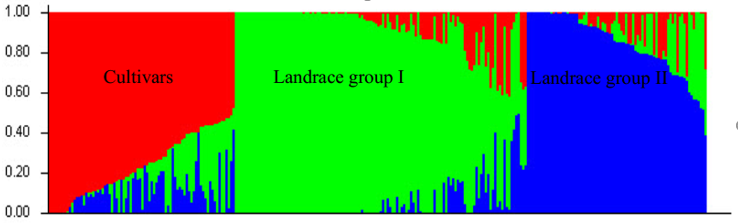


**Fig. S3** Structure plot of 320 Iranian bread wheat accessions determined by K=3 (A) and Principal component analysis (B).


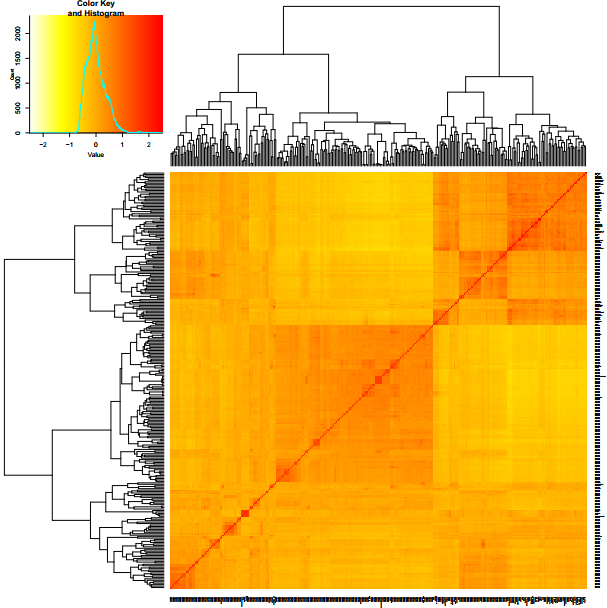


**Varieties**

**Landraces**

**Landraces**

**Fig. S4** Cluster analysis using kiniship matrix for Iranian wheat accessions.
